# Frequency spectrum of chemical fluctuation: A probe of reaction mechanism and dynamics

**DOI:** 10.1371/journal.pcbi.1007356

**Published:** 2019-09-16

**Authors:** Sanggeun Song, Gil-Suk Yang, Seong Jun Park, Sungguan Hong, Ji-Hyun Kim, Jaeyoung Sung

**Affiliations:** 1 Center for Chemical Dynamics in Living Cells, Chung-Ang University, Seoul, Korea; 2 Department of Chemistry, Chung-Ang University, Seoul, Korea; Rutgers University, UNITED STATES

## Abstract

Even in the steady-state, the number of biomolecules in living cells fluctuates dynamically, and the frequency spectrum of this chemical fluctuation carries valuable information about the dynamics of the reactions creating these biomolecules. Recent advances in single-cell techniques enable direct monitoring of the time-traces of the protein number in each cell; however, it is not yet clear how the stochastic dynamics of these time-traces is related to the reaction mechanism and dynamics. Here, we derive a rigorous relation between the frequency-spectrum of the product number fluctuation and the reaction mechanism and dynamics, starting from a generalized master equation. This relation enables us to analyze the time-traces of the protein number and extract information about dynamics of mRNA number and transcriptional regulation, which cannot be directly observed by current experimental techniques. We demonstrate our frequency spectrum analysis of protein number fluctuation, using the gene network model of luciferase expression under the control of the *Bmal 1a* promoter in mouse fibroblast cells. We also discuss how the dynamic heterogeneity of transcription and translation rates affects the frequency-spectra of the mRNA and protein number.

## Author summary

Recent advances in single-cell experimental techniques enable direct visualization of dynamic fluctuations of the biomolecular concentration in each cell; however, a robust, quantitative understanding of the stochastic dynamics of the chemical noise in living cells has yet to be achieved. To understand how the frequency spectrum of product number fluctuation is related to the topology of the reaction network and the dynamics of elementary processes composing the network, we derived an exact analytic result for the frequency spectrum of the product number fluctuation starting from a generalized master equation, enabling the extraction of the frequency spectrum of the reaction rate fluctuation (FSRR) from the frequency spectrum of the product number fluctuation (FSPN). The FSRR serves as a sensitive probe of the mechanism and dynamics of the product creation process; the FSRR vanishes when product creation is a Poisson process. We demonstrated our approach to frequency spectrum analysis of chemical fluctuation for generalized enzyme kinetic models, the gene network model of luciferase expression under the *Bmal 1a* promoter in mouse fibroblast cells, and a more general *vibrant* gene network model.

## Introduction

Fluctuation in the number of chemical species is ubiquitous and particularly pronounced in small reactors such as living cells. This chemical fluctuation persists even in the steady-state, and its stochastic dynamics carries valuable information about the mechanism and the dynamics of the reaction processes that produce the fluctuation. Modern single molecule fluorescence imaging techniques enable direct monitoring of the protein number time-traces in each individual cell [1–6]. A great deal of research has focused on the *magnitude* of protein or mRNA number variation among genetically identical cells [7–9]. However, investigation into the *dynamics* of the protein or mRNA number fluctuation has not been as common [10, 11].

There have been a few pioneering works on the dynamics of chemical fluctuation in living cells. For example, using the chemical master equation (CME), Bratsun, Volfson, Tsimring, and Hasty investigated the protein number frequency spectrum when the decay rate, or the gene expression rate, at a given time is dependent on the protein density at an earlier time due to feedback regulation [12]. McKane, Nagy, Newman and Stefanini investigated the mechanism for pronounced biochemical oscillations based on CME [13]. The CME provides an accurate description of the stochastic chemical dynamics of conventional kinetic network models characterized by constant rate coefficients.

The other approach to understanding the dynamics of chemical fluctuation in living cells is the Gillespie’s chemical Langevin equation (CLE) approach [14]. Using this approach, Ozbudak *et al*. investigated the frequency spectrum of protein number fluctuation for a simple gene expression network model [15]. Simpson, Cox, and Saylor extended this approach to investigate the effects of feedback regulation on the protein number frequency spectrum for the first-order reaction system. [16–18]. Tănase-Nicola, Warren, and Wolde presented a relationship between the frequency spectra of the input and output signals for various biochemical networks [19], starting from the CLE of biochemical networks. Thomas *et al*. obtained the nonlinear correction to the frequency spectrum under the linear-noise approximation for the chemical fluctuation in the vicinity of the Hopf bifurcation point, and explained the oscillatory protein luminescence data obtained for fibroblast cells [20].

The stochasticity of the chemical kinetics based on the CME has been investigated since 1950s. The CME for a first-order reaction was suggested by Bartholomay [21], and CMEs for several types of reaction were reviewed by McQuarrie [22]. However, CME itself is not easy to be solved so that in early days, Kramers [23] and Moyal [24] developed a Taylor expansion of the CME, and takes only the first- and second-order derivative terms, resulting in the chemical Fokker-Planck equation. On the basis of the Poisson representation method, Gardiner and Chaturvedi derived the higher-order generalization of Fokker-Planck equation and the corresponding stochastic differential equation [25]. Separately, van Kampen developed the systematic perturbative expansion of CME, known as system-size expansion [26, 27]. Taking only the leading-order term of the expansion yields the linear-noise approximation, in which the CME can be approximated by a Fokker-Planck equation with linear coefficients [28]. This approach has been widely used in both chemical [13, 29–33] and non-chemical systems [34–42], which is exact up to the second-order moments of chemical fluctuations for any reaction system composed of zeroth- and first-order reactions.

The accuracy of the chemical Fokker-Planck equation or the CLE against the CME is investigated by Grima, Thomas, and Straube [43]. They showed that the chemical Fokker-Planck equation and the corresponding CLE provide a more accurate description than the linear-noise approximation of the CME. Although, in general, the CLE associated with the CME involves the non-Gaussian white noise [44], the widely-used CLE with the Gaussian white noise or various approximations of CME provides a more convenient stochastic description of conventional kinetic network models [15, 45].

Despite these theoretical developments and their prevalence in quantitative biology, it is not feasible for conventional kinetic network models to represent reaction networks in living cells accurately. This is because, for an intracellular reaction process, the rate coefficient may not be a constant but a stochastic variable whose value differs from cell to cell and fluctuates over time due to its coupling to cell environments. Rate coefficient fluctuation also emerges for intracellular reactions that are not simple Poisson processes, for example, multi-step and multi-channel processes involving multiple intermediate species and multiple reaction paths. Consequently, using conventional kinetic network models, it is extremely difficult to achieve a quantitative explanation of stochastic dynamics or its frequency spectrum of biomolecular concentration in living cells [46].

To overcome this problem, a few research groups introduced a static distribution of rate coefficients within the framework of the CME, for example, normal distribution [47, 48], negative binomial distribution [49], and lognormal distribution [50] (see also ref [45] for a recent review). However, the rate coefficient of an intracellular reaction is intrinsically a dynamic stochastic variable that not only differs from cell to cell but also fluctuates over time. Thus, using those approaches, it is difficult to capture the effect of dynamically heterogeneous cell environment on stochastic kinetics in living cells. On the other hand, Sung and Silbey presented the CME for a non-Poisson reaction process occurring under a dynamically heterogeneous environment and an exact master equation for a reactive continuous time random walker undergoing a chemical reaction at a boundary with an arbitrary reaction time distribution [51]. Pedraza and Paulsson investigated how the effect of non-exponential reaction time distributions can be incorporated into the framework of the CME [52] but their approach requires infinite complications for arbitrary reaction time distribution as noted by the authors. Zechner and Koeppl provided the so-called uncoupled network representation in which the effect of fluctuating environment is accounted for by hierarchically coupled moments for the environmental process conditioned on the entire history of the reaction network in interest [53].

For a general representation of reaction processes occurring in living cells, a new type of kinetic network model, called a *vibrant* kinetic network model, was recently introduced [54]. A key feature of vibrant kinetic network models is a stochastic rate coefficient, whose properties depend on the reaction dynamics and its coupling to cell environments. By using a vibrant gene expression network, Park *et al*. obtained the Chemical Fluctuation Theorem governing gene expression [55], which provides a unified, quantitative explanation of the mean and variance of the gene expression level among a clonal population of cells for various experimental systems. In the current work, we investigate the *dynamics* of chemical fluctuation for elementary models of *vibrant* reaction processes, presenting an exact relationship between the frequency spectrum of product number fluctuation and the mechanism and dynamics of the reaction process in question.

We find that the frequency-spectrum of the reaction rate (FSRR) can be easily calculated from the product number time traces and serves as a sensitive probe of the reaction mechanism and dynamics; for example, the FSRR is a monotonically decaying function of frequency for a multi-channel process and a non-monotonic function with one or more peaks for a multi-step reaction process, whereas the FSRR vanishes for a single channel Poisson reaction process. By applying our theory to the translation process, during which proteins are synthesized by ribosomes according to genetic information delivered by *messenger* RNA (mRNA), we extract the frequency spectrum or the time-correlation function of the mRNA number from the time traces of the protein number. This is significant because current experimental techniques enable us to monitor the protein number time traces in each cell but not the mRNA number time traces, or frequency spectra of the mRNA number fluctuation. From the frequency spectrum of the mRNA number fluctuation, we can further extract information about the gene-regulating promoter dynamics. We first demonstrate our frequency spectrum analysis of protein number time traces for the classical gene expression network model utilized by Naef and co-workers [56] to investigate the time traces of luciferase expressed under the control of *Bmal 1a* in mouse fibroblast cells. Then, we investigate the frequency spectra of the mRNA and protein number for a more general gene expression network model, in which promotor regulation and transcription are non-Poisson processes, or *vibrant* reaction processes, that cannot be accurately represented by a classical kinetic network model. Throughout our investigation, we confirm the correctness of our theory against accurate, stochastic simulation.

## Results/discussion

### General theory

The frequency-spectrum of product number (FSPN), *S*_*z*_(*ω*), is defined as

$$S_{z}(\omega )\equiv \underset{T\to \infty }{\lim}T^{-1}\langle {\left|\int_{-T/2}^{T/2}dte^{-i\omega t}\delta z(t)\right|}^{2}\rangle ,$$ (1)

where 〈⋯〉 and *δz*(*t*) denote the average over a large number of trajectories of the product number and the deviation of the product number from the mean at time *t*, respectively. According to the Wiener-Khinchin theorem [57, 58], the frequency-spectrum defined in Eq 1 is equal to the Fourier transform of the steady-state TCF of the product number fluctuation, i.e.,

$$S_{z}(\omega )=\int_{-\infty }^{\infty }dte^{-i\omega t}{\langle \delta z(t)\delta z(0)\rangle }_{ss}.$$ (2)

The functional form of the FSPN, or the TCF of the product creation rate, is dependent on the mechanism and dynamics of the product creation process. Starting from a generalized master equation accurately describing a vibrant reaction process with an arbitrary stochastic rate, one can derive the following analytic result for the FSPN,

$$S_{z}(\omega )=\frac{2\langle R\rangle }{\omega^{2}+\gamma^{2}}+\frac{S_{R}(\omega )}{\omega^{2}+\gamma^{2}},$$ (3)

where 〈*R*〉, *γ*, and *S*_*R*_(*ω*) respectively denote the mean product creation rate, the inverse lifetime of the product molecule, and the frequency-spectrum of the reaction rate (FSRR), that is, $S_{R}(\omega )=\int_{-\infty }^{\infty }dte^{-i\omega t}{\langle \delta R(t)\delta R(0)\rangle }_{ss}$. See Supporting Information, S1 Text for the derivation of Eq 3. This equation holds exactly for vibrant reaction networks free of feedback regulation as long as the decay of the product molecule obeys the first order kinetics. The first term on the right-hand side (R.H.S.) of Eq 3 has the same functional form, regardless of the detailed mechanism and dynamics of the product creation process; this term is determined by only two parameters: the mean product creation rate and the inverse lifetime of the product molecule. In contrast, the second term involving the frequency spectrum, *S*_*R*_(*ω*), of the reaction rate fluctuation, or the Fourier transform of the TCF of the reaction rate fluctuation, is dependent on the topology of the product creation network and the dynamics of the individual reaction processes that compose the network.

Although the CLE is only approximately valid, we can also obtain Eq 3 by using the CLE under the following ad-hoc assumptions of the reaction rate fluctuation: 1) the fluctuating rate can be represented by the sum of intrinsic and extrinsic noise, which are independent of each other; 2) intrinsic noise is white noise whose variance is the same as the mean total reaction rate or the sum of the mean intrinsic and extrinsic rate; 3) only extrinsic noise is dependent on the details of the reaction mechanism and dynamics and their coupling to environment while intrinsic noise is not. However, starting from our generalized master equation, Eq 3 can be derived without these ad-hoc assumptions or approximations, as shown in S1 Text in Supporting Information.

The frequency-spectrum of the reaction rate, *S*_*R*_(*ω*), can be easily extracted from the FSPN, *S*_*z*_(*ω*). From Eq 3, one obtains

$$S_{R}(\omega )=\left(\omega^{2}+\gamma^{2}\right)\left[S_{z}(\omega )-S_{z}^{0}(\omega )\right]=2\langle R\rangle \left[S_{z}(\omega )/S_{z}^{0}(\omega )-1\right],$$ (4)

where $S_{z}^{0}(\omega )$ denotes 2〈*R*〉/(*ω*^2^+*γ*^2^), the first term on the R.H.S. of Eq 3. Given that the inverse lifetime, *γ*, of product molecules can be estimated independently, we can easily calculate the mean product creation rate, 〈*R*〉, from the mean product number, 〈*z*〉, by 〈*R*〉 = *γ*〈*z*〉. With 〈*R*〉 and *γ* at hand, we can calculate $S_{z}^{0}(\omega )$ and convert the FSPN, *S*_*z*_(*ω*), to the FSRR, *S*_*R*_(*ω*), by using Eq 4.

**Fig 1:**
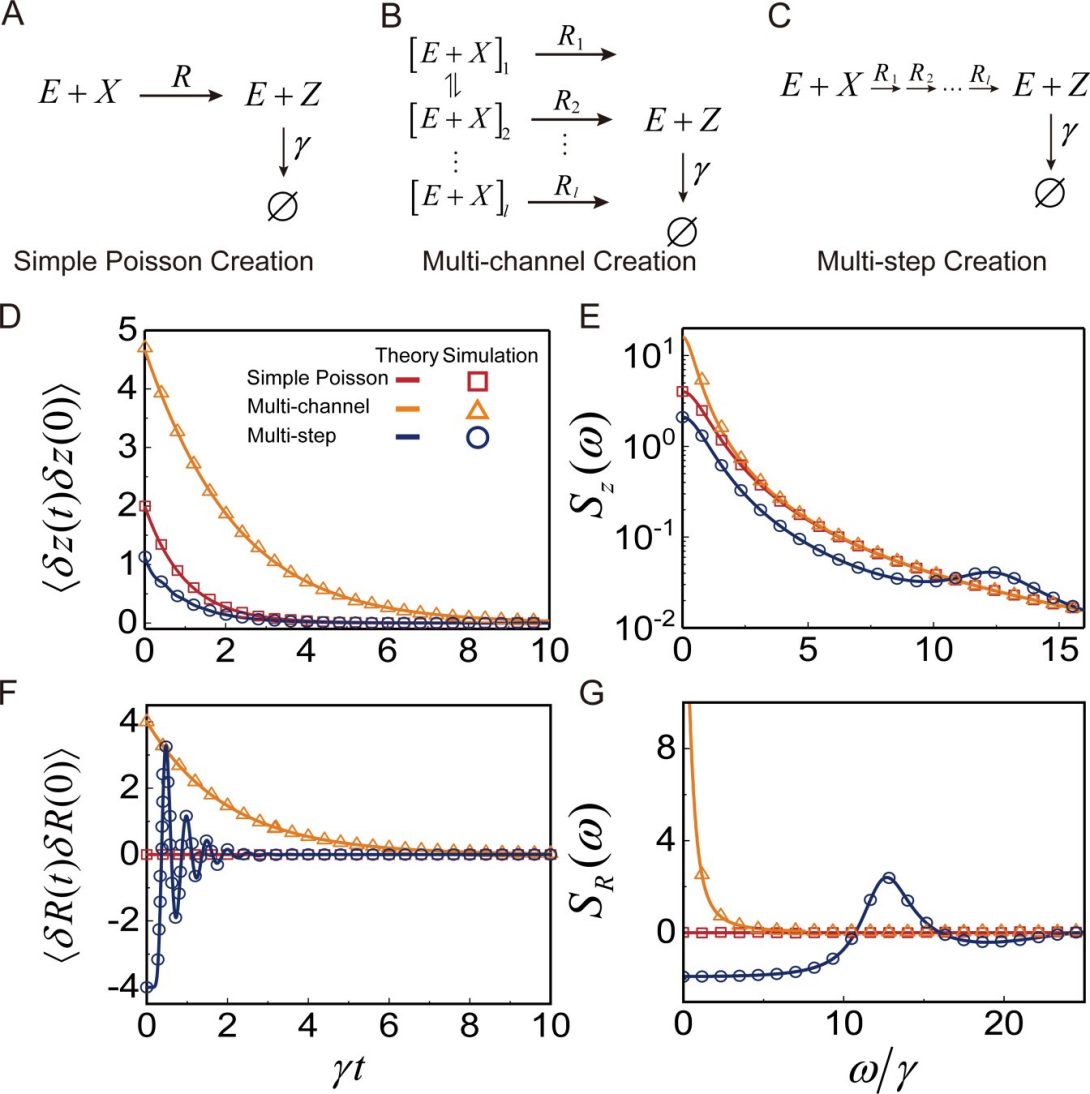
Time autocorrelation functions and frequency spectra of the product number and the product creation rate for three elementary reaction networks. (A-C) Reaction schemes for the three different types of product creation processes: the simple Poisson process, the multi-channel process, and the multi-step process. The product decay process is a simple Poisson process. (D-E) Time autocorrelation function of the product number fluctuation and the frequency spectrum of the product number (FSPN) for the reaction schemes (A-C). (F-G) Time autocorrelation function of the product creation rate fluctuation and the frequency spectrum of the product creation rate (FSRR). The FSRR is related to the FSPN by Eq 4. The FSRR is far more sensitive to changes in the reaction scheme than the FSPN. The FSRR is zero at all frequencies for Scheme A, a monotonically decaying function for Scheme B, and a non-monotonic function for Scheme C. In the model calculation, the mean time elapsed during product creation is set to 0.5 *γ*^−1^ for all cases. The solid lines represent the predictions of Eq 3 for Scheme A, Scheme B with *l* = 2, and Scheme C with *l* = 20. Simulation results are represented by squares, triangles, and circles. For more detailed information about the models and simulation method, see Supporting Information, S3 Text.

The FSRR, *S*_*R*_(*ω*), can be easily obtained for various reaction network models. For example, in Fig 1, we show *S*_*R*_(*ω*) and the corresponding TCF of the product creation rate for three different reaction processes: the simple one-step process, the multi-channel process, and the multi-step process. In the simplest case, where the product creation reaction is a simple Poisson process, the product creation rate is constant in time, so that 〈*δR*(*t*)*δR*(0)〉 = *S*_*R*_(*ω*) = 0. When the product creation process is a multi-channel reaction process, shown in Fig 1B, the TCF of the product creation rate is given by a multi-exponential function of time, and the corresponding FSRR is a monotonically decaying function of frequency, *ω* (see Eqs S2-5 and S2-6 in Supporting Information). In contrast, for a multi-step reaction process, shown in Fig 1C, the TCF of the product creation rate becomes an oscillatory function of time as the step number increases, so that the FSRR is a non-monotonic function with one or more peaks (see Eq S2-12). The oscillatory feature in the TCF of the product creation rate can be understood from the degradation-free mean product number, 〈*n*(*t*)〉*, under the synchronized initial condition that the reaction event counting begins at the time when a reaction event is completed [55, 59]. The TCF of the product creation rate is related to 〈*n*(*t*)〉* as 〈*δR*(*t*)*δR*(0)〉 = 〈*R*〉∂_*t*_〈*n*(*t*)〉^*^− 〈*R*〉^2^ [55], enabling to calculate the rate correlation with 〈*n*(*t*)〉^*^ directly obtained from simulations as shown in Fig 1F. As the number, *l*, of steps involved in the creation process increases, reaction times are more narrowly distributed around the mean reaction time, 〈*t*〉(= 〈*R*〉^−1^); in the large-*l* limit, the reaction time distribution approaches a Dirac delta function given by *δ*(*t*−〈*t*〉). When reaction events occur with more precise timing, 〈*n*(*t*)〉^*^ increases with time in a more step-like manner. Then, the first-order time derivative of 〈*n*(*t*)〉^*^ shows a more distinct oscillatory feature over time so that the TCF of the product creation rate also does. The derivation of the exact analytic expressions of the FSRR, *S*_*R*_(*ω*), are presented for both the multi-channel reaction process and the multi-step reaction process in Supporting Information, S2 Text.

We confirm the correctness of our analytic results against stochastic simulation results, as shown in Fig 1D–1G. In this way, we can double check both analytical results and simulation results at once, making them more convincing against each other. For each model in Fig 1A–1C, the FSPN can be computed directly from simulated product number time traces using the fast Fourier transform algorithm by Eq 1 (Supporting Information, S3 Text). This FSPN, $S_{z}^{\left(\mathrm{sim}\right)}(\omega )$, is in perfect agreement with *S*_*z*_(*ω*) calculated using Eq 3 and the analytic results of FSRR, *S*_*R*_(*ω*), given in Supporting Information, S2 Text for all three reaction models (Fig 1E). Accordingly, for each reaction model, the FSRR *S*_*R*_(*ω*) calculated from the analytic result is in perfect agreement with the FSRR data, $S_{R}^{\left(\mathrm{data}\right)}(\omega )$, obtained from the numerical data of $S_{z}^{\left(\mathrm{sim}\right)}(\omega )$ through Eq 4, as shown in Fig 1G. We also confirm that FSRR calculated from the TCF of the product number using Eq 2, yields the same result as FSRR directly calculated from the product number trajectories using Eq 1.

Both the theoretical prediction and the simulation results indicate that the frequency spectrum of the reaction rate is a more sensitive probe of the dynamics of the product creation process than the frequency spectrum of the product number, as shown in Fig 1. This is because $S_{z}^{0}(\omega )$, which is independent of the microscopic details of the product creation process, always contributes to the FSPN, *S*_*z*_(*ω*), whereas the FSRR, *S*_*R*_(*ω*), is not contributed from $S_{z}^{0}(\omega )$.

### Application to non-classical enzyme kinetic models

**Fig 2:**
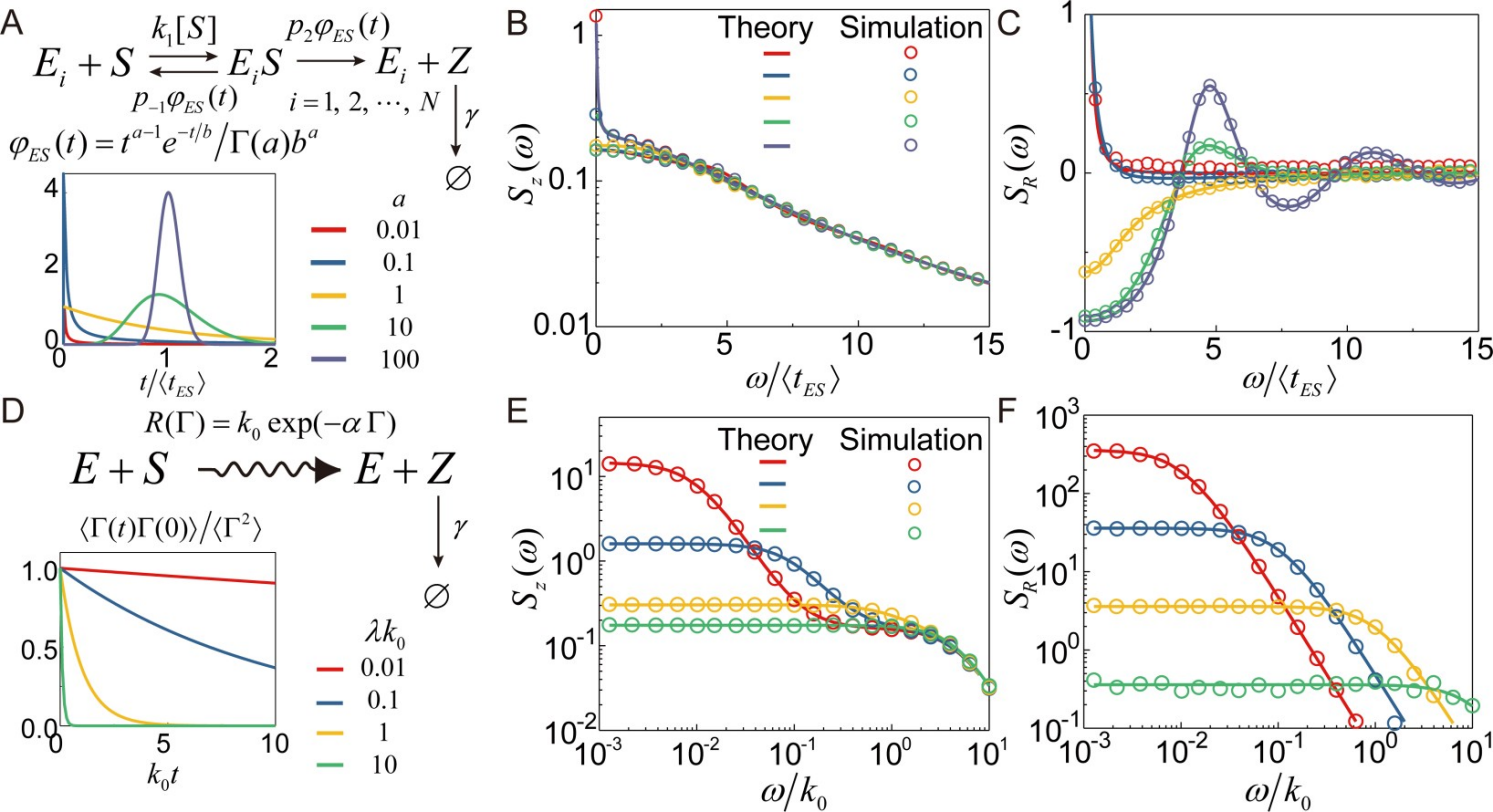
Frequency spectra of the product number and the product creation rate for two enzymatic reaction models. (A) A multi-enzyme reaction scheme for product creation. Individual reaction processes of *N* identical enzymes are independent of each other. A bimolecular enzyme-substrate association occurs at a rate of *k*_1_[*S*] with [*S*] denoting the substrate concentration. *φ*_*ES*_(*t*) represents the lifetime distribution of an enzyme-substrate complex (*ES*), which is modeled as a gamma distribution, i.e. *φ*_*ES*_(*t*) = *t*^*a*−1^*e*^−*t*/*b*^/Γ(*a*)*b*^*a*^. *p*_2,−1_ are the probabilities associated to catalytic reaction and enzyme-substrate dissociation. (inset) *φ*_*ES*_(*t*) for various values of *a*. 〈*t*_*ES*_〉 is the mean lifetime of an *ES* complex defined by $\langle t_{ES}\rangle =\int_{0}^{\infty }dt\;t\varphi_{ES}(t)$. (B-C) Theoretical predictions (solid lines) and simulation results (circles) for the FSPN and FSRR for Scheme a with *k*_1_〈*t*_*ES*_〉[*S*] = 1 and *p*_2_ = *p*_−1_ = 1/2. The number of single enzymes is 10. Different colors indicate different values of *a*. (D) A vibrant catalytic reaction scheme for product creation by a single enzyme. The catalytic reaction is set to be the rate determining step in Scheme A, so that the entire enzymatic reaction rate can be represented by the catalytic reaction rate, *R*(Γ). The fluctuation of *R*(Γ) due to coupling with a hidden variable, Γ, is modeled as an exponential model, *R*(Γ) = *k*_0_*e*^−*α*Γ^. (inset) The time correlation function of Γ, which is here modelled as an Ornstein-Uhlenbeck process. The fluctuation rate of Γ is given by *λ*. (E-F) Theoretical predictions (solid lines) and simulation results (circles) for the FSPN and FSRR for Scheme D for various values of *λ*. The value of *k*_0_/*κ*_0_ is chosen to be 0.64. The product decay process is a simple Poisson process. The value of *γ* is given by *γ*〈*t*_*ES*_〉 = 5 in (B) and *γ* = 5*k*_0_ in (C). For more detailed information about the models and simulation method, see Methods.

The generalized enzyme kinetic model shown in Fig 2A is remarkable because it quantitatively explains experimental results for the substrate concentration-dependent variance of *β*-galactosidase’s turnover time distribution [60, 61], which cannot be explained by the classical Michaelis-Menten enzyme kinetics or its extension considering a static distribution of the catalytic rate [8]. The key feature of this generalized enzyme kinetic model is that the catalytic reaction and dissociation reaction of the enzyme-substrate (ES) complex are non-Poisson processes so that the lifetime distribution, *φ*_*ES*_(*t*), of the ES complex is a non-exponential function. For this generalized enzyme reaction model, the enzymatic reaction time distribution, *ψ*(*t*), or the distribution of time elapsed to complete a single enzymatic turnover, is given by

$$\tilde{\psi }(\omega )=\frac{p_{2}{\tilde{\varphi }}_{1}(\omega ){\tilde{\varphi }}_{ES}(\omega )}{1-p_{-1}{\tilde{\varphi }}_{1}(\omega ){\tilde{\varphi }}_{ES}(\omega )}$$ (5)

with ${\tilde{\varphi }}_{1}(\omega )=k_{1}[S]/\left(k_{1}[S]+i\omega \right)$, where $\tilde{f}(\omega )$ designates $\int_{0}^{\infty }dte^{-i\omega t}f(t)$. Eq 5 can be obtained from the generalized enzyme kinetics in refs [60, 62] and also starting from Sung and Silbey’s generalized master equation [63]. However, it is not so easy to obtain Eq 5 using the conventional CME, which requires infinite complications when the enzyme-substrate complex has an arbitrary lifetime distribution as noted by the authors of ref [52]. The ES formation has been often assumed to be the pseudo-first order reaction [64], which may not always be the case in living cells. The extension of our enzyme reaction model to encompass the second-order kinetics of the ES formation is also possible [65, 66], which we leave for a future research.

The enzyme reaction process represented by Fig 2A is an example of a renewal process [60, 61], in which the dynamics of one reaction event is not affected by the reaction history. For a renewal reaction process, the FSRR is related to the reaction time distribution, *ψ*(*t*), by

SR(ω)=2〈R〉Re[ψ˜(ω)1−ψ˜(ω)]−2π〈R〉2δ(ω), (6)

where 〈*R*〉 denotes the average reaction rate given by $\langle R\rangle =\lim_{\omega \to 0}\left(i\omega )\tilde{\psi }(\omega \right)/[1-\tilde{\psi }(\omega )]$. A simple derivation of Eq 6 is given in Supporting Information, S4 Text. Substituting Eq 5 into Eq 6, we obtain the frequency spectrum of the reaction rate fluctuation for the generalized enzyme reaction model in Fig 2A. Substituting Eq 6 into Eq 3, we can further obtain the frequency spectrum of the product number fluctuation. As shown in Fig 2B and 2C, the frequency spectrum of the rate fluctuation is far more sensitive to the reaction dynamics, or the lifetime distribution of the enzyme-substrate complex, than the frequency spectrum of the product number fluctuation.

It is worth mentioning that the reaction process of multiple enzymes is not a renewal process, even when the reaction process of individual enzyme is [59, 67]. Nevertheless, given that the correlation between different enzymes is negligible, the FSRR, $S_{R}^{\left(n\right)}(\omega )$, of *n* enzymes is simply given by $nS_{R}^{\left(1\right)}(\omega )$, where $S_{R}^{\left(1\right)}(\omega )$ denotes the FSRR of a single enzyme reaction, given by Eq 6.

We emphasize that the application range of Eqs 3 and 4 is not limited to only renewal reaction processes. A simple example of a non-renewal process is a catalytic reaction of enzymes whose reaction rate varies depending on the enzyme’s conformation. In Fig 2D, we present a simple model of a non-renewal enzymatic process with a single rate-determining step with activation energy, *E*_*a*_, weakly coupled to an enzyme conformation coordinate, *r*. Applying the Arrhenius equation to this model, one can easily obtain the following formula for the reaction rate: *R* = *A*exp(−*βE*_*a*_(*r*))≅*k*_0_ exp(−*α*Γ) with *k*_0_ = *A*exp[−*βE*_*a*_(*r*_*eq*_)], $\alpha =\beta {\partial E_{a}(r)/\partial r]}_{r=r_{eq}}$, and Γ = *r*−*r*_*eq*_, where *r*_*eq*_ denotes the value of *r* at the equilibrium conformation of the enzyme. When Γ(*t*) is a stationary Gaussian process, this model is exactly solvable, and the time correlation function of the reaction rate is simply given by

$$\langle R(t)R(0)\rangle =k_{0}^{2}\exp\left[\alpha^{2}\langle \Gamma^{2}\rangle \right]\exp\left[\alpha^{2}\langle \Gamma (t)\Gamma (0)\rangle \right].$$ (7)

The reaction represented by this model is a non-renewal process because individual reaction events occur with different reaction rates and the individual reaction times are correlated with each other. It is easy to extend the Gillespie algorithm to simulate this simple vibrant reaction model (See Methods). In Fig 2E, we present the FSPN, $S_{z}^{\left(\mathrm{sim}\right)}(\omega )$, calculated by applying Eq 1 to the product number trajectories obtained by stochastic simulation of this reaction model. Using Eq 4, we can convert the FSPN, $S_{z}^{\left(\mathrm{sim}\right)}(\omega )$, into the FSRR, $S_{R}^{\left(\mathrm{sim}\right)}(\omega )$. As shown in Fig 2F, this FSRR obtained from the simulation result is in excellent agreement with our theoretical results, $S_{R}^{}(\omega )=\int_{-\infty }^{\infty }dte^{i\omega t}\langle R(t)R(0)\rangle$ with the TCF given in Eq 7. Substituting this result of FSRR, *S*_*R*_(*ω*), into Eq 3, we obtain the theoretical result of the FSPN. As shown in Fig 2E, this exact theoretical result of the FSPN is also in agreement with $S_{z}^{\left(\mathrm{sim}\right)}(\omega )$ obtained from the simulation results, demonstrating the correctness of our theory and our stochastic simulation algorithm. Again, both theory and simulation make it clear that the FSRR is far more sensitive to the enzyme conformation dynamics than the FSPN.

An important example of a non-renewal process is the gene expression process; rates of the chemical processes constituting gene expression can be a stochastic variable depending on various cell-state variables, such as the promoter-regulation state, the population of gene machinery proteins and transcription factors, the phase in the cell cycle, and the nutrition state, forcing the stochastic property of gene expression to deviate from a simple renewal process [54, 68].

### Application to a conventional gene expression network model

**Fig 3:**
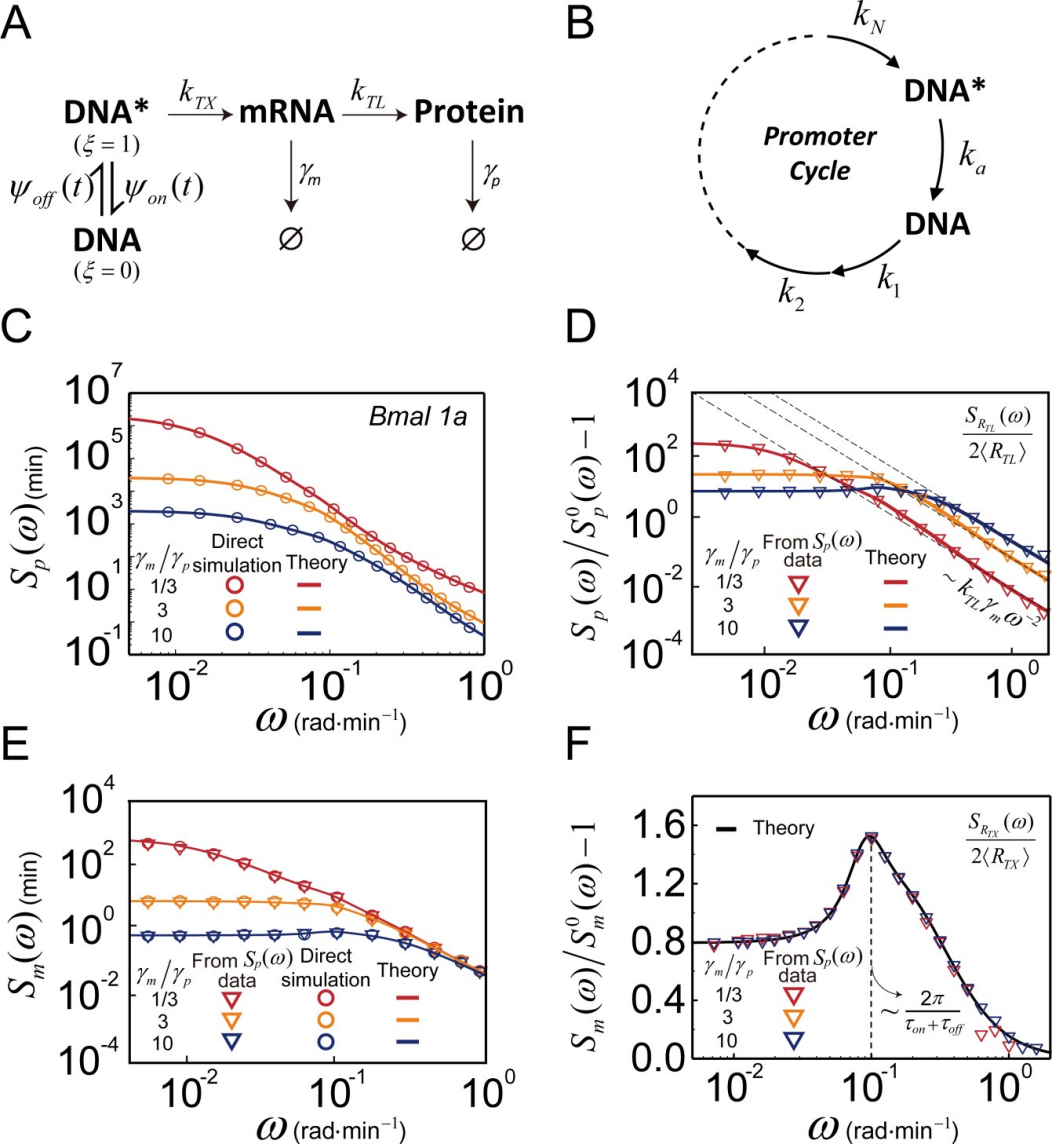
Spectral analysis of protein number fluctuation. (A) Reaction network model of *luciferase* expression controlled by the *Bmal 1a* promoter in mouse fibroblast cells [56]. *ψ*_*on*_(*t*) and *ψ*_*off*_(*t*) represent the lifetime distribution of the active and inactive gene states, respectively. (B) Model of the gene deactivation and activation cycle of the *Bmal 1a* promoter. Gene deactivation is a simple Poisson process, but gene activation is a non-Poisson process composed of *N* consecutive Poisson processes. (C) Frequency spectrum of protein number at three different ratios of the mRNA lifetime to the protein lifetime. (circle) simulation results; (lines) theoretical results. (D) Modified frequency spectrum of protein number or mean-scaled frequency spectrum of translation rate, $S_{p}(\omega )/S_{p}^{0}(\omega )-1\left[=S_{R_{TL}}(\omega )/2\langle R_{TL}\rangle \right]$. $S_{p}^{0}(\omega )$ is defined as $S_{p}^{0}(\omega )=2\langle R\rangle /\left(\omega^{2}+\gamma_{p}^{2}\right)$. In the high frequency regime, the asymptotic behavior of the modified frequency spectrum is given by *k*_*TL*_*γ*_*m*_*ω*^−2^, i.e., $\lim_{\omega \to \infty }\omega^{2}\left[S_{p}(\omega )/S_{p}^{0}(\omega )-1\right]=k_{TL}\gamma_{m}$. (E) Frequency spectrum of the mRNA number. (triangles) data extracted from the frequency spectrum data, *S*_*p*_(*ω*), of the protein number with use of Eq 8. (circles) simulation results, (lines) the theoretical results of Eqs 3 and 10. (F) Modified mRNA number frequency spectrum or mean-scaled frequency spectrum of transcription rate. (triangles) data extracted from *S*_*p*_(*ω*) with use of Eqs 8 and 9. (line) the theoretical result of Eq 10.

We demonstrate an application of Eqs 3 and 4 to a quantitative analysis of the time traces of the protein copy number for the gene expression network model shown in Fig 3A and 3B. This model was used by Naef and coworkers to investigate the time traces of the number of luciferase expressed under the *Bmal 1a* promoter in mouse fibroblast cells [56]. According to this model, the transcription process under the *Bmal 1a* promoter involves a sub-Poisson gene activation process composed of 7 intermediate reaction steps and a simple one-step gene deactivation process, a Poisson process [56, 69], as schematically represented in Fig 3B. For this gene expression network model, we conduct a stochastic simulation to obtain the time traces of the protein copy number, and then use these time traces to calculate the protein number frequency spectrum with Eq 1. We compare this frequency spectrum with the theoretical prediction of Eq 3 and FSRR of the same gene expression network model (see Supporting Information, S5 Text). As shown in Fig 3C, the prediction of Eq 3 is in perfect agreement with the simulation results. An explicit, analytic result of the protein number frequency spectrum obtained from Eq 3 is presented in Supporting Information, S5 Text.

We can obtain the mRNA number frequency spectrum, *S*_*m*_(*ω*), from the protein number frequency spectrum, *S*_*p*_(*ω*), by applying Eq 4 to the translation process. This is noteworthy because the mRNA number frequency spectrum, or the mRNA number time trace, is difficult to obtain using currently available experimental tools. In the gene expression network model shown in Fig 3A, the protein creation rate, or the translation rate, is given by *R*_*TL*_ = *k*_*TL*_*m*, where *k*_*TL*_ and *m* are the translation rate coefficient and the mRNA copy number, respectively. In general, *k*_*TL*_ represents the translation rate per mRNA and is a stochastic variable dependent on various cell state variables, which include ribosome concentration, concentrations of amino acids, and mRNA conformation to name a few. However, let us first consider the simplest case where, compared to the mRNA number variation, the fluctuation of *k*_*TL*_ negligibly influences the protein number fluctuation, which is found to be true in bacterial gene expression [1]. By applying Eq 4 to the translation process, we obtain

$$k_{TL}^{2}S_{m}(\omega )=(\omega^{2}+\gamma_{p}^{2})\left[S_{p}(\omega )-S_{p}^{0}(\omega )\right]=2\langle R_{TL}\rangle \left[S_{p}(\omega )/S_{p}^{0}(\omega )-1\right],$$ (8)

where $S_{p}^{0}(\omega )=2\langle R_{TL}\rangle /\left(\omega^{2}+\gamma_{p}^{2}\right)$. As mentioned above, 〈*R*_*TL*_〉(= *k*_*TL*_〈*m*〉) can be estimated from the mean protein level and the inverse lifetime of protein, that is, 〈*R*_*TL*_〉 = 〈*p*〉*γ*_*p*_ or *k*_*TL*_ = 〈*p*〉*γ*_*p*_/〈*m*〉. Given that the inverse lifetime, *γ*_*m*_, of mRNA can be estimated separately, the value of *k*_*TL*_ can also be estimated from the following asymptotic relation, $S_{p}^{}(\omega )/S_{p}^{0}(\omega )-1≅k_{TL}\gamma_{m}\omega^{-2}$, valid in the high frequency regime (see Supporting Information, S5 Text). Even in the presence of the strong cell-to-cell variation in the translation rate coefficient, the relation between the mRNA number frequency spectrum and protein number frequency spectrum is similar to Eq 8, as shown later in this work.

For the gene expression network model in Fig 3A, we can obtain the mRNA number frequency spectrum, *S*_*m*_(*ω*), by three different methods: first, using Eq 8 with protein number power spectrum obtained by applying Eq 1 or 2 to the stochastic simulation results of the protein number time traces, as described in the previous paragraph, second, using Eq 3 with the exact analytic expression of the TCF of the transcription rate, available to this model (see Eq S5-9 in Supporting Information), and third, using numerical simulation of the mRNA number trajectories to calculate the frequency spectrum of the mRNA number fluctuation by Eq 1 or 2. As shown in Fig 3E, all three results are in excellent agreement.

The frequency spectrum of mRNA number fluctuation can be converted to the frequency spectrum of the transcription rate fluctuation, which is difficult to observe experimentally. Applying Eq 4 to the transcription process, we convert the mRNA number frequency spectrum to the frequency spectrum of the transcription rate, i.e.,

$$S_{R_{TX}}(\omega )=\left(\omega^{2}+\gamma_{m}^{2}\right)\left[S_{m}(\omega )-S_{m}^{0}(\omega )\right]=2\langle R_{TX}\rangle \left[S_{m}(\omega )/S_{m}^{0}(\omega )-1\right],$$ (9)

where $S_{m}^{0}(\omega )$ is defined as $S_{m}^{0}(\omega )=2\langle R_{TX}\rangle /\left(\omega^{2}+\gamma_{m}^{2}\right)$ with 〈*R*_*TX*_〉 = 〈*m*〉*γ*_*m*_. As shown in Fig 3F, the frequency spectrum of the transcription rate fluctuation, extracted from the mRNA number frequency spectrum, is independent of mRNA lifetime, consistent with the model in Fig 3A where transcription and mRNA decay are not correlated, while the mRNA number frequency spectrum is dependent on mRNA lifetime. Note that the frequency spectrum of the transcription rate, obtained by Eq 9, and the mRNA number frequency spectrum, calculated by simulation trajectories of the mRNA number, are in good agreement with the prediction of our analytic result for the gene expression network model, which is given in the next paragraph.

The frequency spectrum of the transcription rate fluctuation carries valuable information about the dynamics of the transcription regulation process. For the transcription network model shown in Fig 3A, the transcription rate can be represented by *R*_*TX*_ = *ξk*_*TX*_. Here, *ξ* denotes the stochastic variable representing the promoter regulating gene state, whose value is 1 for the gene in the active state but 0 for the gene in the inactive state, and *k*_*TX*_ denotes the active gene transcription rate. For the model shown in Fig 3A, the frequency spectrum of the transcription rate is related to the lifetime distribution, *ψ*_*on*(*off*)_(*t*), of the active (inactive) gene state by [70]

SRTX(ω)=kTX2Sξ(ω)=2kTX2τon+τoffRe[1−ψ^on(s)][1−ψ^off(s)]s2[1−ψ^on(s)ψ^off(s)]|s=iω. (10)

Here, ${\hat{\psi }}_{on(off)}(s)$ and ${\hat{\psi }}_{on(off)}(s)$ denote the frequency spectrum of the gene state variable, *ξ*, and the Laplace transform of the lifetime distribution of the active (inactive) gene state, respectively. *τ*_*on*(*off*)_ designates the mean lifetime of the active (inactive) gene state, that is, $\tau_{on(off)}^{}=\int_{0}^{\infty }dt\;t\psi_{on(off)}(t)$.

The non-monotonic frequency spectrum of the transcription rate, shown in Fig 3F, emerges when the gene activation process is a multi-step consecutive reaction process. For the gene activation-deactivation model shown in Fig 3B, the deactivation of the active gene is a Poisson process, and *ψ*_*on*_(*t*) is a simple exponential function; in contrast, the gene activation process is a multi-step process with *ψ*_*off*_(*t*) being a non-monotonic, unimodal distribution (see Supporting Information, S6 Text). For this model, ${\hat{\psi }}_{on}(s)$ and ${\hat{\psi }}_{off}(s)$ are given by *k*_*a*_/(*s*+*k*_*a*_) and $\prod_{i=1}^{N}k_{i}/\left(s+k_{i}\right)\left[\equiv \hat{f}(k,s)\right]$, respectively. According to reference [56], the activation process of *Bmal 1a* in the embryonic stem cells of mice is best represented by 7 consecutive Poisson reaction processes, and the corresponding lifetime distribution, *ψ*_*off*_(*t*), of the inactive gene state involves 7 different rate parameters, the optimized values of which are given by *k*_1_ = *k*_2_ = ⋯ = *k*_6_ = 9.93×10^−2^min^−1^ and *k*_7_ = 0.23min^−1^. Substituting these expressions of ${\hat{\psi }}_{on}(s)$ and ${\hat{\psi }}_{off}(s)$ into Eq 10, we obtain the explicit analytic result of $S_{R_{TX}}(\omega )$ for the classical gene expression network model in Fig 3A. We find that *f*(**k**,*t*), the inverse Laplace transform of $\hat{f}(k,s)$, with these optimized parameter values can be approximated by a gamma distribution, *t*^*a*−1^*e*^−*t*/*b*^/*b*^*a*^Γ(*a*) (Supporting Information, S7 Text).

### Application to a *vibrant* gene expression network model

**Fig 4:**
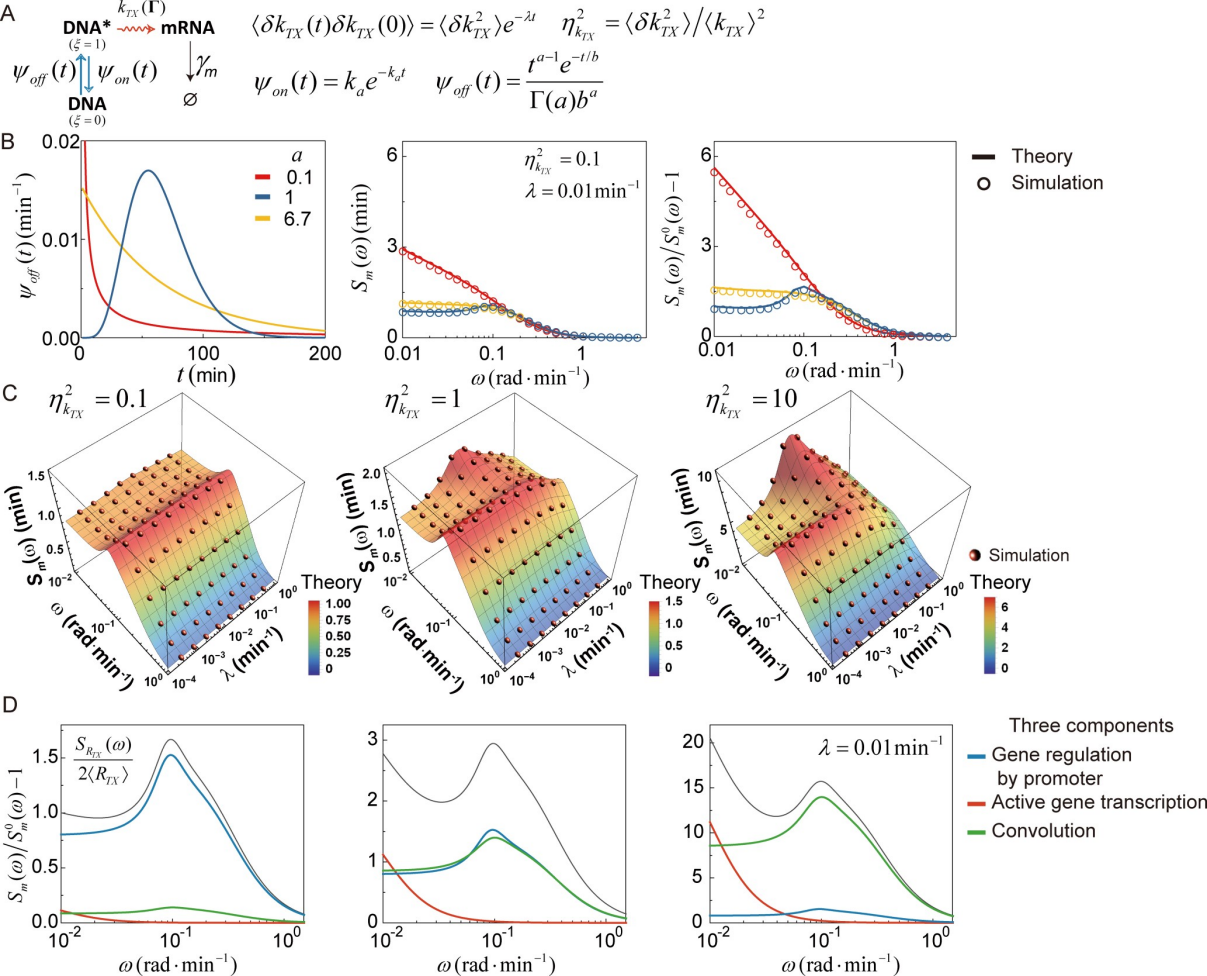
mRNA number frequency spectrum of gene expression network model with super-Poisson active gene transcription dynamics. (A) Gene expression network model with the *vibrant* active gene transcription process. The active gene transcription rate, *k*_*TX*_, is a dynamic, stochastic variable. The mean value, 〈*k*_*TX*_〉, of the active gene transcription rate is the same as the value of *k*_*TX*_ in Fig 3. The normalized time correlation function (TCF) of *k*_*TX*_ is given by an exponentially decaying function of time, i.e., $\phi_{k_{TX}}(t)=\exp(-\lambda t)$. An example of a reaction scheme with the exponential TCF of the rate fluctuation is a two-channel reaction model (see Fig 1B and 1F). Gene regulation by the promoter is modeled the same as Model II in Fig 3B. (B) Dependence of the mRNA number frequency spectrum (middle) and the mean-scaled frequency spectrum of the transcription rate (right) on the lifetime distributions of inactive gene state with the same mean but different shape parameters (left). The values of the relaxation rate, *λ*, and the relative variance, $\eta_{k_{TX}}^{2}$, of the fluctuation of *k*_*TX*_ are set to be 0.01 min^-1^and 0.1, respectively. (lines) The theoretical results for the mRNA number frequency spectrum calculated by Eqs 3 and 11. (circles) Stochastic simulation results (see Supporting Information, S8 Text). (C) Dependence of the mRNA number frequency spectrum on *λ* for three different values of $\eta_{k_{TX}}^{2}$. (surface) The theoretical result. (spheres) Stochastic simulation results. The mRNA number frequency spectrum has a peak around the promoter fluctuation frequency *ω*_*peak*_ = 2*π*/(*τ*_*on*_+*τ*_*off*_)≅0.1 rad∙min^−1^, originating from the sub-Poisson gene regulation dynamics of the promoter. The peak diminishes when $\eta_{k_{TX}}^{2}$ is large and *k*_*TX*_ fluctuates quickly. (D) Three components of the frequency spectrum of the transcription rate. (black line) the value of $S_{R_{TX}}(\omega )/\left(2\langle R_{TX}\rangle \right)\left[=\langle R_{TX}\rangle {\tilde{S}}_{R_{TX}}(\omega )/2\right]$ or $S_{m}^{}(\omega )/S_{m}^{0}(\omega )-1$ with $S_{m}^{0}(\omega )=2\left(\langle m\rangle \gamma_{m}\right)/\left(\omega^{2}+\gamma_{m}^{2}\right)$. The value of *λ* is set to be 0.01 min^−1^. (colored lines) three components of the frequency spectrum: *S*_*ξ*_(*ω*) originating from the gene regulating dynamics of the promoter (blue), $S_{k_{TX}}(\omega )$ originating from the active gene transcription dynamics (red), and their convolution $S_{\xi }(\omega )*S_{k_{TX}}(\omega )$ (green). See Eq 11. The relative contribution of $S_{k_{TX}}(\omega )$ and $S_{\xi }(\omega )*S_{k_{TX}}(\omega )$ increases with $\eta_{k_{TX}}^{2}$, while *S*_*ξ*_(*ω*) does not. When the value of $\eta_{k_{TX}}^{2}$ is 10, the relative contribution of *S*_*ξ*_(*ω*) is marginal, but the non-monotonic peak of the frequency spectrum persists due to the contribution of the convolution term, $S_{\xi }(\omega )*S_{k_{TX}}(\omega )$, given that *λ* is smaller than *ω*_*peak*_ (see S3 Fig for the case where *λ* is as large as *ω*_*peak*_).

Although active gene transcription and translation of each mRNA are often assumed to be simple Poisson processes in gene expression models in the literature, these processes can be complicated, non-Poisson processes, because they are composed of multi-step or multi-channel elementary processes and because their reaction rates may be coupled to heterogeneous cell environments [54]. With this in mind, from this point forward, we consider the mRNA and protein number frequency spectra of the more general transcription network model shown in Fig 4A, where gene activation is a non-Poisson renewal process and transcription of active genes is a vibrant reaction process with the rate being a dynamic stochastic variable, which differs from cell to cell and fluctuates over time.

The mRNA number frequency spectrum of the transcription network shown in Fig 4A is dependent on both the dynamics of gene regulation and active gene transcription dynamics. For this model, the transcription rate can be represented by *R*_*TX*_ = *ξk*_*TX*_(Γ). Here, *ξ* and *k*_*TX*_(Γ) denote the gene state variable defined above Eq 10 and the active gene transcription rate that is dependent on cell state variables, Γ. The frequency spectrum, $S_{R_{TX}}(\omega )$, of the transcription rate for the model shown in Fig 4A is obtained as

$${\tilde{S}}_{R_{TX}}(\omega )={\tilde{S}}_{\xi }(\omega )+{\tilde{S}}_{k_{TX}}(\omega )+{\tilde{S}}_{\xi }(\omega )*{\tilde{S}}_{k_{TX}}(\omega ),$$ (11)

where ${\tilde{S}}_{q}(\omega )$ denotes *S*_*q*_(*ω*)/〈*q*〉^2^ (see Supporting Information, S8 Text for the derivation of Eq 11). In Eq 11, ${\tilde{S}}_{\xi }(\omega )$ is determined by the microscopic dynamics of the gene regulation processes related to the lifetime distributions of the on and off states by S˜ξ(ω)=(τon+τoff)2Sξ(ω)/τon2=2(τon+τoff)/τon2Re[1−ψ^on(s)][1−ψ^off(s)]/s2[1−ψ^on(s)ψ^off(s)]|s=iω. On the other hand, ${\tilde{S}}_{k_{TX}}(\omega )$ in Eq 11 is dependent on the transcription dynamics of the gene in the active state. We consider a vibrant, active gene-transcription model with a stochastically fluctuating rate whose time correlation function is an exponential function. Simple examples of such vibrant reaction models include Zwanzig’s enzyme reaction model [71], whose rate-determining step is a product escape process from the enzyme’s active site through a fluctuating channel, and a two-state enzyme reaction model with a state-dependent dichotomous reaction rate. For this model, ${\tilde{S}}_{k_{TX}}(\omega )$ is a monotonically decaying function of frequency (see Fig 1G for an example), given by ${\tilde{S}}_{k_{TX}}(\omega )=\lambda {\left(\omega^{2}+\lambda^{2}\right)}^{-1}\eta_{k_{TX}}^{2}$ with *λ* and $\eta_{k_{TL}}^{2}$ denoting the relaxation rate, defined by $\langle \delta k_{TX}(t)\delta k_{TX}(0)\rangle =\langle \delta k_{TX}^{2}\rangle e^{-\lambda t}$, and the relative variance of the active gene transcription rate fluctuation, respectively. As shown in Fig 4B, for all cases investigated, the mRNA number frequency spectrum, calculated by Eq 3 and Eq 11, is in excellent agreement with the spectrum obtained by simulated mRNA number trajectories.

The frequency spectrum of the transcription rate fluctuation is more sensitive to dynamics of gene regulation than the frequency spectrum of the mRNA number fluctuation. The frequency spectrum of the transcription rate fluctuation is a monotonically decaying function of frequency when the gene-activation process is a Poisson or super-Poisson process [72]; however, this spectrum shows a non-monotonic frequency dependence when gene activation is a strongly sub-Poisson process, as shown in Fig 4B.

The frequency spectrum of the transcription rate fluctuation is also sensitive to the magnitude and speed of the active gene transcription rate fluctuation. As can be seen in Fig 4C, when $\eta_{k_{TL}}^{2}=0.1$, the frequency spectrum, ${\tilde{S}}_{R_{TX}}(\omega )$, of the transcription rate is a non-monotonic function of frequency, nearly identical to Fig 3F, and shows no strong dependence on the relaxation speed, *λ*, of the active gene transcription rate fluctuation. This is because, when $\eta_{k_{TL}}^{2}<<1$, ${\tilde{S}}_{\xi }(\omega )$, originating from gene regulation of the promoter, is the major contributor to ${\tilde{S}}_{R_{TX}}(\omega )$, while ${\tilde{S}}_{k_{TX}}(\omega )$, originating from active gene transcription, is only a minor contributor (see Fig 4C). However, as $\eta_{k_{TX}}^{2}$ increases, so too does the contribution from the active gene transcription dynamics to $S_{R_{TX}}(\omega )$, causing $S_{R_{TX}}(\omega )$ to significantly deviate from *S*_*ξ*_(*ω*); $S_{R_{TX}}(\omega )$ is a monotonically decaying function of frequency in the low frequency regime due to the contribution from $S_{k_{TX}}(\omega )$, as demonstrated in Fig 4B and 4C. When $\eta_{k_{TX}}^{2}$ is far greater than unity, ${\tilde{S}}_{R_{TX}}(\omega )$ is dominantly contributed from ${\tilde{S}}_{k_{TX}}(\omega )$ and ${\tilde{S}}_{\xi }(\omega )*{\tilde{S}}_{k_{TX}}(\omega )$, the last two terms on the R.H.S. of Eq 11, and it is due to the latter contribution that ${\tilde{S}}_{R_{TX}}(\omega )$ has a non-monotonic frequency dependency in contrast to ${\tilde{S}}_{k_{TX}}(\omega )$.

### Generalizations

Although we have so far assumed the translation rate coefficient, *k*_*TL*_, is constant, it can also be a random variable, the value of which differs from cell to cell. We find that, for this case as well, Eq 3 holds and the protein number frequency spectrum is related to the mRNA number frequency spectrum by

$$S_{p}(\omega )/S_{p}^{0}(\omega )-1=\langle k_{TL}\rangle (1+\eta_{k_{TL}}^{2})S_{m}(\omega )/2\langle m\rangle ,$$ (12)

as long as the cell-to-cell heterogeneity of *k*_*TL*_ is much greater than the dynamic fluctuation of *k*_*TL*_ in each cell (see Supporting Information, S10 Text).

Gene copy number variation is another factor that potentially affects the frequency spectrum of mRNA number or protein number fluctuation. Naef and co-workers investigated the gene regulation dynamics of the *Bmal 1a* promoter in mouse fibroblast cells that do not differentiate. For this system, the gene copy number is always unity and does not vary. However, in general, gene copy number varies with time in a time scale much longer than the individual transcription event. In the simplest case, where the correlation between the number of proteins created by one gene and the number of proteins created by another is negligible, the frequency spectrum of the protein number is given by the frequency spectrum of the protein number of a single gene system multiplied by the average gene copy number. However, the expression levels of two non-interacting genes are correlated because of shared environment effects. We leave it to future research to investigate how the cell-environment induced correlation between the expression levels of different genes affects the frequency spectrum of mRNA and protein.

Both mRNA decay and protein decay are enzyme reaction processes that may differ greatly from simple Poisson processes. Additionally, in the presence of feedback gene regulation, the gene expression rate is dependent on the protein number. For such cases, Eq 3 and the equations that are derived from Eq 3 in this work are only approximately valid. A generalization of this work to encompass these cases is possible and will appear elsewhere.

We finish this section by emphasizing that, in application of our theory to the analysis of experimental FSPN data, accuracy of the FSRR extracted from the FSPN data using Eq 4 relies on the accuracy of the FSPN data. That is to say, to obtain correct information about the reaction mechanism and dynamics from the FSRR, one first has to obtain accurate FSPN data from which the FSRR is extracted. This can be experimentally challenging, especially when the mean reaction rate, 〈*R*〉, is far greater than the FSRR, *S*_*R*_(*ω*), or when the first term on the R.H.S. of Eq 3 is far greater than the second term.

### Conclusion

We investigated how the frequency spectrum of product number fluctuation is related to the topology of the reaction network and the dynamics of elementary processes composing the network. For this purpose, we derived an exact analytic result for the frequency spectrum of the product number fluctuation starting from a generalized master equation. This result enables one to obtain the frequency spectrum of the reaction rate fluctuation (FSRR) from the frequency spectrum of the product number fluctuation (FSPN). The FSRR is more sensitive to the mechanism and dynamics of the product creation process than the FSPN. The FSRR vanishes when product creation is a Poisson process. However, the FSRR is a monotonically decaying function of frequency, when the product creation process is a super-Poisson process, such as a multi-channel process, but is a non-monotonic function of frequency with one or more peaks when the reaction is a sub-Poisson process, such as a multi-step process.

Our theory is applicable not only to the conventional kinetic network model but also to vibrant reaction network model consisting of multistep and/or multichannel elementary processes with arbitrary reaction orders, reaction time distributions, and rate coefficient fluctuations. Vibrant gene expression network models enables quantitative understanding of the mRNA and protein number fluctuations for various gene expression systems [54, 55], which could not be quantitatively explained by the conventional gene network models and the CME. An advantage of our approach is that we don’t have to construct *a priori* explicit model for the environment coupled to the system network; we explicitly model only the control variable dependent part of the entire network and the effects of the remaining part of the network and environment are collectively accounted for by the time correlation function (TCF) of the rate fluctuation. This vibrant reaction network model based approach is useful in quantitative analysis of chemical fluctuations generated from intracellular reaction networks consisting of elementary reaction processes with arbitrary reaction time distribution and environment coupled rate fluctuations, for which it is a difficult to construct the correct and explicit model in terms of the conventional kinetic network model consisting of a few discrete chemical states and Poisson transition process between them.

We demonstrated our approach to frequency spectrum analysis of chemical fluctuation for a generalized enzyme kinetic models showing that the frequency spectrum of reaction rate serves as a sensitive probe of the reaction dynamics of the enzyme-substrate complex. Then, by applying our approach to a gene expression network, we can extract the mRNA number frequency spectrum from the protein number frequency spectrum. From the mRNA number frequency spectrum, we can further extract quantitative information about the gene regulation dynamics of the promoter and the active gene transcription dynamics. This was demonstrated for the gene network model of *luciferase* expression under the *Bmal 1a* promoter in mouse fibroblast cells and for a more general *vibrant* gene network model.

## Methods

In this section, we present the detailed algorithm used to generate the simulation results in Fig 2. For the enzymatic reaction model [61, 68] in Fig 2A, which is more general than the conventional Michaelis-Menten model, every stochastic trajectory begins with the enzyme-substrate association step (*E*+*S*→*ES*). The time elapsed for each enzyme-substrate association event (*E*+*S*→*ES*) is sampled from $\varphi_{1}(t)=k_{1}[S]e^{-tk_{1}[S]}$. The lifetime of an ES complex is then sampled from a non-exponential distribution, here, the gamma distribution, *φ*_*ES*_(*t*) = *t*^*a*−1^*e*^−*t*/*b*^/Γ(*a*)*b*^*a*^. The fate of a given ES complex, that is, either dissociation (*E*+*S*←*ES*) or catalytic reaction (*ES*→*E*+*P*), is chosen using the probability, *p*_2_, of catalytic reaction. A uniform random number is then generated between 0 and 1, and if it is smaller than *p*_2_ at that time, a catalytic reaction occurs, resulting in a product molecule. Otherwise, the ES complex is dissociated into a free enzyme and a substrate. Either case is followed by another round of enzyme-substrate association reactions. The lifetime of each product molecule is sampled from $\gamma_{z}e^{-t\gamma_{z}}$.

To obtain the FSPN of the *N*-enzyme reaction system considered in Fig 2A, *N* single-enzyme trajectories, independent of each other, are simultaneously generated and superposed to yield a single trajectory of the product number fluctuation. Because we need stationary pooled trajectories to calculate the FSPN, the initial time of pooled trajectories is arbitrarily chosen at a time long enough that the distribution of the product number reaches its stationary state.

For vibrant reaction models, the reaction rate, *R*(**Γ**), stochastically fluctuates over time because of its coupling to state variable **Γ**, which can be a multidimensional vector. When a stochastic realization of **Γ** is generated with a time step, Δ*t*, the reaction probability, *R*(**Γ**(*t*))Δ*t*, is calculated at every time step and compared with a uniform random number between 0 and 1. When *R*(**Γ**(*t*))Δ*t* is less than this random number, a reaction occurs, resulting in a product molecule at that time.

Our method generalizes the algorithm in reference [73] to a state-dependent, non-Poisson reaction process. Lim et al.’s algorithm [73] is valid when the fluctuation time scale of **Γ** is much longer than the time scale of the individual reaction event. However, the current method is free of such limitation. In comparison with the Extrande method developed by Voliotis, Thomas, Grima, and Bowsher [74], our method shows a faster convergence rate under the same condition (see S5 Fig). In Fig 2B, the conformation coordinate, Γ, of an enzyme is modelled as a simple Ornstein-Uhlenbeck process characterized by zero mean, unit variance, and relaxation rate, *λ*, of the exponentially decaying time correlation function, 〈Γ(*t*)Γ(0)〉/〈Γ^2^〉 = *e*^−*tλ*^. We use the following update equation to generate this process: $\Gamma (t+\Delta t)=\Gamma (t)e^{-\lambda \Delta t}+{\left(1-e^{-2\lambda \Delta t}\right)}^{1/2}N[0,1]$ with N[0,1] denoting a Gaussian random number with zero mean and unit variance [75]. For Γ to be a stationary process from the beginning of the simulation, every trajectory of Γ must begin with Γ(t=0)=N[0,1]. The reaction rate, *R*(Γ), is modelled as *R*(Γ) = *k*_0_*e*^−*α*Γ^, with *k*_0_ and *α* being constant in time, and the value of Δ*t* is chosen to be *k*_0_Δ*t* = 10^−3^ here.

## Supporting information

S1 Text Derivation of Eq 3. (PDF)

S2 Text Power spectrum of the product creation rate for multi-channel and multi-step processes. (PDF)

S3 Text Simulation method for Fig 1. (PDF)

S4 Text Derivation of Eq 6. (PDF)

S5 Text Analytic expressions for power spectra in Fig 3. (PDF)

S6 Text Simulation method for Fig 3. (PDF)

S7 Text Power spectrum analysis for approximated *ψ*_*off*_(*t*). (PDF)

S8 Text Analytic expression for power spectrum of the transcription rate in Fig 4. (PDF)

S9 Text Simulation method for Fig 4. (PDF)

S10 Text Derivation of Eq 12. (PDF)

S1 Fig Power spectrum analysis of *Bmal 1a* + TSA gene expression. (PDF)

S2 Fig Quantitative analysis of the protein number frequency spectrum. (PDF)

S3 Fig Three components in the power spectrum of the transcription rate. (PDF)

S4 Fig Effect of the cell-to-cell heterogeneity in translation rate coefficient, *k*_*TL*_, on the protein number power spectrum, *S*_*p*_(*ω*). (PDF)

S5 Fig Performance of the current method of simulating reaction events occurring with stochastically fluctuating rate in comparison with the Extrande method. (PDF)
