# Supplementary material for: Frequency spectrum of chemical fluctuation: A probe of reaction mechanism and dynamics
Source: PLoS Comput Biol. 2019 Sep 16;15(9):e1007356. doi: 10.1371/journal.pcbi.1007356 (PMC6762214; doi:10.1371/journal.pcbi.1007356)
Supplement: S1 Text — (PDF) [file pcbi.1007356.s001.pdf]

Supplementary Text 1 | Derivation of Eq 3.

In this section, we provide a detailed derivation of Eq 3, one of the key results in this work. First, we consider the Fourier transform of the product number time autocorrelation function, given on the right-hand side (R.H.S) of Eq 2. Using the Euler's formula, $e^{i\theta} = \cos\theta + i\sin\theta$, we can write the Fourier transform as

$$\int_{-\infty}^{\infty} dte^{-i\omega t} \langle \delta z(t)\delta z(0)\rangle_{ss} = \int_{-\infty}^{\infty} dt \cos(\omega t)\langle \delta z(t)\delta z(0)\rangle_{ss} - i\int_{-\infty}^{\infty} dt \sin(\omega t)\langle \delta z(t)\delta z(0)\rangle_{ss} , \tag{S1-1}$$

which is known as the cosine and sine transform. In the steady-state, the time correlation function of product number, $\langle \delta z(t)\delta z(0)\rangle_{ss}$, is an even function, that is, $\langle \delta z(-t)\delta z(0)\rangle_{ss} = \langle \delta z(t)\delta z(0)\rangle_{ss}$, so that the first term on the R.H.S as

$$\int_{-\infty}^{\infty} dt \cos(\omega t)\langle \delta z(t)\delta z(0)\rangle_{ss} = 2\,\mathrm{Re}\left[\int_{0}^{\infty} dte^{-i\omega t}\langle \delta z(t)\delta z(0)\rangle_{ss}\right], \tag{S1-2}$$

and the second term on the R.H.S vanishes. Recalling the definition of the Laplace transform, the R.H.S of the above equation is same as the Laplace transform of $\langle \delta z(t)\delta z(0)\rangle_{ss}$, where the Laplace variable is equal to $i\omega$. When we denote the Laplace transform of the product number time autocorrelation function as $\hat{\varphi}_z(s) \equiv \int_0^{\infty} dte^{-st}\langle \delta z(t)\delta z(0)\rangle_{ss}$, the relationship between the power spectrum of the product number satisfies

$$S_z(\omega) = 2\,\mathrm{Re}[\hat{\varphi}_z(i\omega)] = \hat{\varphi}_z(i\omega) + \hat{\varphi}_z(-i\omega). \tag{S1-3}$$

To derive the expression of $\hat{\varphi}_z(s)$ for an intracellular birth-death process, where the product creation rate is coupled to cell state variables, we consider the following generalized master equation (GME) [1, 2]'

$$\frac{\partial}{\partial t}p_z(\mathbf{\Gamma},t)=R(\mathbf{\Gamma})[p_{z-1}(\mathbf{\Gamma},t)-p_z(\mathbf{\Gamma},t)]+\gamma[(z+1)p_{z+1}(\mathbf{\Gamma},t)-zp_z(\mathbf{\Gamma},t)]+L(\mathbf{\Gamma})p_z(\mathbf{\Gamma},t)\,, \tag{S1-4}$$

where $p_z(\mathbf{\Gamma},t)$ is the probability that z number of products exist at cell state $\mathbf{\Gamma}$ at time t. $R(\mathbf{\Gamma})$ denotes the product creation rate dependent on the cell state variable, and $L(\mathbf{\Gamma})$ designates the time evolution operator of cell state variable, $\mathbf{\Gamma}$. The steady-state time correlation function (TCF) of the product number is given by

$$\langle z(t)z(0)\rangle_{ss}=\int d\mathbf{\Gamma}_0\sum_{z_0=0}^{\infty}\langle\overline{z}(t\,|\,z_0,\mathbf{\Gamma}_0)\rangle z_0 p_{ss}(z_0,\mathbf{\Gamma}_0)\quad, \tag{S1-5}$$

where $\langle\overline{z}(t\,|\,z_0,\mathbf{\Gamma}_0)\rangle$ and $p_{ss}(z_0,\mathbf{\Gamma}_0)$ respectively denote the average number of the product molecules at time t, given that the product number is given by z_0 and the cell state is at $\mathbf{\Gamma}_0$ at time 0, and the joint distribution function of the product number and the cell state variable in the steady-state. The mathematical definition of $\langle\overline{z}(t\,|\,z_0,\mathbf{\Gamma}_0)\rangle$ is given by $\langle\overline{z}(t\,|\,z_0,\mathbf{\Gamma}_0)\rangle\equiv\int d\mathbf{\Gamma}\sum_{z=0}^{\infty}zp_z(\mathbf{\Gamma},t\,|\,z_0,\mathbf{\Gamma}_0)$, where $p_z(\mathbf{\Gamma},t\,|\,z_0,\mathbf{\Gamma}_0)$ denotes the solution of Eq S1-4 under the following initial condition, $p_z(\mathbf{\Gamma},t=0)=\delta_{zz_0}\delta(\mathbf{\Gamma}-\mathbf{\Gamma}_0)$. To obtain $\langle\overline{z}(t\,|\,z_0,\mathbf{\Gamma}_0)\rangle$, let us first obtain the time-evolution equation for $\overline{z}(\mathbf{\Gamma},t\,|\,z_0,\mathbf{\Gamma}_0)$, or $\sum_{z=0}^{\infty}zp_z(\mathbf{\Gamma},t\,|\,z_0,\mathbf{\Gamma}_0)$, the integration of which over $\mathbf{\Gamma}$ yields $\langle\overline{z}(t\,|\,z_0,\mathbf{\Gamma}_0)\rangle$. From Eq S1-4, we obtain

$$\frac{\partial}{\partial t}\overline{z}(\mathbf{\Gamma},t\,|\,z_0,\mathbf{\Gamma}_0)=R(\mathbf{\Gamma})p(\mathbf{\Gamma},t\,|\,z_0,\mathbf{\Gamma}_0)-\gamma\overline{z}(\mathbf{\Gamma},t\,|\,z_0,\mathbf{\Gamma}_0)+L(\mathbf{\Gamma})\overline{z}(\mathbf{\Gamma},t\,|\,z_0,\mathbf{\Gamma}_0)\,, \tag{S1-6}$$

where $p(\mathbf{\Gamma},t\,|\,z_0,\mathbf{\Gamma}_0)$ is defined as $p(\mathbf{\Gamma},t\,|\,z_0,\mathbf{\Gamma}_0)\equiv\sum_{z=0}^{\infty}p_z(\mathbf{\Gamma},t\,|\,z_0,\mathbf{\Gamma}_0)$. By taking the Laplace transform of Eq S1-6, we obtain the following formal expression for the Laplace transform of $\overline{z}(\mathbf{\Gamma},t\,|\,z_0,\mathbf{\Gamma}_0)$:

$$\hat{\overline{z}}(\mathbf{\Gamma},s\,|\,z_0,\mathbf{\Gamma}_0)=\left[s+\gamma-L(\mathbf{\Gamma})\right]^{-1}\left[z_0\delta(\mathbf{\Gamma}-\mathbf{\Gamma}_0)+R(\mathbf{\Gamma})\hat{p}(\mathbf{\Gamma},s\,|\,z_0,\mathbf{\Gamma}_0)\right]. \qquad \text{(S1-7)}$$

To obtain this equation, we have used the following initial condition, $\overline{z}(\mathbf{\Gamma},0\,|\,z_0,\mathbf{\Gamma}_0)=\sum_{z=z_0}^{\infty}z\delta_{zz_0}\delta(\mathbf{\Gamma}-\mathbf{\Gamma}_0)=z_0\delta(\mathbf{\Gamma}-\mathbf{\Gamma}_0)$. In terms of the Green's function defined by $\hat{G}(\mathbf{\Gamma},s\,|\,\mathbf{\Gamma}_0)=\left[s-L(\mathbf{\Gamma})\right]^{-1}\delta(\mathbf{\Gamma}-\mathbf{\Gamma}_0)$, we can rewrite Eq S1-7 as follows:

$$\hat{\overline{z}}(\mathbf{\Gamma},s\,|\,z_0,\mathbf{\Gamma}_0)=z_0\hat{G}(\mathbf{\Gamma},s+\gamma\,|\,\mathbf{\Gamma}_0)+\left[s+\gamma-L(\mathbf{\Gamma})\right]^{-1}R(\mathbf{\Gamma})\hat{p}(\mathbf{\Gamma},s\,|\,z_0,\mathbf{\Gamma}_0). \qquad \text{(S1-8)}$$

Our next task is to find the expression for $p(\mathbf{\Gamma},t\,|\,z_0,\mathbf{\Gamma}_0)\left[\equiv\sum_{z=0}^{\infty}p_z(\mathbf{\Gamma},t\,|\,z_0,\mathbf{\Gamma}_0)\right]$, whose Laplace transform appears on the R.H.S. of Eq S1-8. By applying $\sum_{z=0}^{\infty}(\cdots)$ on both sides of Eq S1-4, we obtain the evolution equation of $p(\mathbf{\Gamma},t\,|\,z_0,\mathbf{\Gamma}_0)$ as follows:

$$\frac{\partial}{\partial t}p(\mathbf{\Gamma},t\,|\,z_0,\mathbf{\Gamma}_0)=L(\mathbf{\Gamma})p(\mathbf{\Gamma},t\,|\,z_0,\mathbf{\Gamma}_0). \qquad \text{(S1-9)}$$

Noting that the initial condition of this equation is given by $p(\mathbf{\Gamma},0\,|\,\mathbf{\Gamma}_0)=\delta(\mathbf{\Gamma}-\mathbf{\Gamma}_0)$, one can show that the solution of Eq S1-9 is actually the same as the Green's function defined above in Eq S1-8, that is, $\hat{p}(\mathbf{\Gamma},s\,|\,z_0,\mathbf{\Gamma}_0)=[s-L(\mathbf{\Gamma})]^{-1}\delta(\mathbf{\Gamma}-\mathbf{\Gamma}_0)=\hat{G}(s,\mathbf{\Gamma}\,|\,\mathbf{\Gamma}_0)$. Substituting the latter equation into Eq S1-8, and using the following identity,

$$\begin{aligned}\left[s+\gamma-L(\mathbf{\Gamma})\right]^{-1}f(\mathbf{\Gamma})&=\left[s+\gamma-L(\mathbf{\Gamma})\right]^{-1}\int d\mathbf{\Gamma}\delta(\mathbf{\Gamma}-\mathbf{\Gamma}_1)f(\mathbf{\Gamma}_1)\\&=\int d\mathbf{\Gamma}_1\hat{G}(\mathbf{\Gamma},s+\gamma\,|\,\mathbf{\Gamma}_1)f(\mathbf{\Gamma}_1),\end{aligned} \qquad \text{(S1-10)}$$

we obtain

$$\hat{\hat{z}}(\mathbf{\Gamma},s\,|\,z_0,\mathbf{\Gamma}_0)=z_0\hat{G}(\mathbf{\Gamma},s+\gamma\,|\,\mathbf{\Gamma}_0)+\int d\mathbf{\Gamma}_1\hat{G}(\mathbf{\Gamma},s+\gamma\,|\,\mathbf{\Gamma}_1)R(\mathbf{\Gamma}_1)\hat{G}(\mathbf{\Gamma}_1,s\,|\,\mathbf{\Gamma}_0)\,. \quad \text{(S1-11)}$$

Taking the integral over $\mathbf{\Gamma}$ on both sides of this equation, we obtain

$$\langle\hat{\hat{z}}(s\,|\,z_0,\mathbf{\Gamma}_0)\rangle=\frac{z_0}{s+\gamma}+\frac{1}{s+\gamma}\int d\mathbf{\Gamma}R(\mathbf{\Gamma})\hat{G}(\mathbf{\Gamma},s\,|\,\mathbf{\Gamma}_0)\,. \quad \text{(S1-12)}$$

In the derivation of Eq S1-12, we have used the following identity, $\int d\mathbf{\Gamma}\hat{G}(\mathbf{\Gamma},s\,|\,\mathbf{\Gamma}_0)=1/s$, which is nothing but the Laplace transform of the normalization condition, i.e., $\int d\mathbf{\Gamma}G(\mathbf{\Gamma},t\,|\,\mathbf{\Gamma}_0)=1$. Substituting Eq S1-12 into the Laplace transform of Eq S1-5, we obtain

$$\begin{aligned}\mathcal{L}[\langle z(t)z(0)\rangle_{ss}]&=\sum_{z_0=0}^{\infty}\int d\mathbf{\Gamma}_0\langle\hat{\hat{z}}(s\,|\,z_0,\mathbf{\Gamma}_0)\rangle z_0p_{ss}(z_0,\mathbf{\Gamma}_0)\\&=\int d\mathbf{\Gamma}_0\sum_{z_0=0}^{\infty}\frac{z_0^2}{s+\gamma}p_{ss}(z_0,\mathbf{\Gamma}_0)\\&+\int d\mathbf{\Gamma}_0\sum_{z_0=0}^{\infty}\frac{z_0}{s+\gamma}\int d\mathbf{\Gamma}R(\mathbf{\Gamma})\hat{G}(\mathbf{\Gamma},s\,|\,\mathbf{\Gamma}_0)p_{ss}(z_0,\mathbf{\Gamma}_0).\end{aligned} \quad \text{(S1-13)}$$

Rearranging the above equation, we find that

$$\mathcal{L}[\langle z(t)z(0)\rangle_{ss}]=\frac{\langle z^2\rangle_{ss}}{s+\gamma}+\frac{1}{s+\gamma}\int d\mathbf{\Gamma}\int d\mathbf{\Gamma}_0R(\mathbf{\Gamma})\hat{G}(\mathbf{\Gamma},s\,|\,\mathbf{\Gamma}_0)\langle z(\mathbf{\Gamma}_0)\rangle_{ss}\,, \quad \text{(S1-14)}$$

where $\langle z^2\rangle_{ss}$ and $\langle z(\mathbf{\Gamma}_0)\rangle_{ss}$ are given by $\int d\mathbf{\Gamma}_0\sum_{z_0=0}^{\infty}z_0^2p_{ss}(z_0,\mathbf{\Gamma}_0)=\sum_{z_0=0}^{\infty}z_0^2p_{ss}(z_0)$ and $\sum_{z_0=0}^{\infty}z_0p_{ss}(z_0,\mathbf{\Gamma}_0)$, respectively. To obtain the expression for $\langle z(\mathbf{\Gamma}_0)\rangle_{ss}$, we should obtain the expression for $p_{ss}(z,\mathbf{\Gamma})$. Due to the fact that, in the steady-state, the probability density satisfying Eq S1-4 does not change over time, we obtain the following equation for $p_{ss}(z,\mathbf{\Gamma})$:

$$R(\mathbf{\Gamma})[p_{ss}(z-1,\mathbf{\Gamma})-p_{ss}(z,\mathbf{\Gamma})]-\gamma(z+1)p_{ss}(z+1,\mathbf{\Gamma})-zp_{ss}(z,\mathbf{\Gamma})+L(\mathbf{\Gamma})p_{ss}(z,\mathbf{\Gamma})=0.$$

(S1-15)

By applying $\sum_{z=0}^{\infty} z$ on both sides of this equation and then rearranging the resulting equation, we can obtain

$$\begin{aligned}\langle z(\mathbf{\Gamma})\rangle_{ss} &= [\gamma - L(\mathbf{\Gamma})]^{-1} R(\mathbf{\Gamma}) p_{ss}(\mathbf{\Gamma}) \\ &= \int d\mathbf{\Gamma}_1 \hat{G}(\mathbf{\Gamma}, \gamma \mid \mathbf{\Gamma}_1) R(\mathbf{\Gamma}_1) p_{ss}(\mathbf{\Gamma}_1).\end{aligned} \tag{S1-16}$$

Substituting Eq S1-16 into Eq S1-14, we obtain

$$\begin{aligned}\mathcal{L}[\langle z(t)z(0)\rangle_{ss}] &= \frac{\langle z^2\rangle_{ss}}{s+\gamma} + \frac{1}{s+\gamma}\int d\mathbf{\Gamma}\int d\mathbf{\Gamma}_0 R(\mathbf{\Gamma})\hat{G}(\mathbf{\Gamma}, s \mid \mathbf{\Gamma}_0)[\gamma - L(\mathbf{\Gamma}_0)]^{-1} R(\mathbf{\Gamma}_0) p_{ss}(\mathbf{\Gamma}_0) \\ &= \frac{\langle z^2\rangle_{ss}}{s+\gamma} + \frac{1}{s+\gamma}\int d\mathbf{\Gamma}\int d\mathbf{\Gamma}_0 R(\mathbf{\Gamma})[s - L(\mathbf{\Gamma})]^{-1}\delta(\mathbf{\Gamma}-\mathbf{\Gamma}_0)[\gamma - L(\mathbf{\Gamma}_0)]^{-1} R(\mathbf{\Gamma}_0) p_{ss}(\mathbf{\Gamma}_0) \\ &= \frac{\langle z^2\rangle_{ss}}{s+\gamma} + \frac{1}{s+\gamma}\int d\mathbf{\Gamma} R(\mathbf{\Gamma})[s - L(\mathbf{\Gamma})]^{-1}[\gamma - L(\mathbf{\Gamma})]^{-1} R(\mathbf{\Gamma}) p_{ss}(\mathbf{\Gamma}).\end{aligned}$$

(S1-17)

Taking advantage of the following operator identity, $A^{-1}B^{-1} = \frac{1}{A-B}(B^{-1} - A^{-1})$, where $A-B$ is a scalar function, we have $[s - L(\mathbf{\Gamma})]^{-1}[\gamma - L(\mathbf{\Gamma})]^{-1}$ = $(s-\gamma)^{-1}\left\{[\gamma - L(\mathbf{\Gamma})]^{-1} - [s - L(\mathbf{\Gamma})]^{-1}\right\}$. Substituting this identity into Eq S1-17, we obtain

$$\begin{aligned}\mathcal{L}[\langle z(t)z(0)\rangle_{ss}] &= \frac{\langle z^2\rangle_{ss}}{s+\gamma} - \frac{1}{s^2-\gamma^2}\int d\mathbf{\Gamma} R(\mathbf{\Gamma})[s - L(\mathbf{\Gamma})]^{-1} R(\mathbf{\Gamma}) p_{ss}(\mathbf{\Gamma}) \\ &+ \frac{1}{s^2-\gamma^2}\int d\mathbf{\Gamma} R(\mathbf{\Gamma})[\gamma - L(\mathbf{\Gamma})]^{-1} R(\mathbf{\Gamma}) p_{ss}(\mathbf{\Gamma}).\end{aligned} \tag{S1-18}$$

In terms of Green's function, $\int d\mathbf{\Gamma} R(\mathbf{\Gamma})[s - L(\mathbf{\Gamma})]^{-1} R(\mathbf{\Gamma}) p_{ss}(\mathbf{\Gamma})$, appearing in Eq S1-18 , can be written as $\int d\mathbf{\Gamma}\int d\mathbf{\Gamma}_0 R(\mathbf{\Gamma})\hat{G}(\mathbf{\Gamma}, s \mid \mathbf{\Gamma}_0) R(\mathbf{\Gamma}_0) p_{ss}(\mathbf{\Gamma}_0)$, and this is nothing but the Laplace transform of the steady-state time correlation function (TCF) of the product creation rate defined by

$$\langle R(t)R(0)\rangle_{ss} \equiv \int d\mathbf{\Gamma}\int d\mathbf{\Gamma}_0 R(\mathbf{\Gamma})G(\mathbf{\Gamma},t\,|\,\mathbf{\Gamma}_0)R(\mathbf{\Gamma}_0)p_{ss}(\mathbf{\Gamma}_0). \tag{S1-19}$$

In terms of the TCF, Eq S1-18 can be written as

$$\mathcal{L}[\langle z(t)z(0)\rangle_{ss}] = \frac{\langle z^2\rangle_{ss}}{s+\gamma} + \frac{1}{s^2-\gamma^2}\left[\int_0^\infty dt e^{-\gamma t}\langle R(t)R(0)\rangle - \int_0^\infty dt e^{-st}\langle R(t)R(0)\rangle\right]. \tag{S1-20}$$

Subtracting $\langle z\rangle_{ss}^2/s$ from the both sides of Eq S1-20, and using the following identities, $\langle z\rangle_{ss} = \langle R\rangle/\gamma$ and $\langle R(t)R(0)\rangle = \langle \delta R(t)\delta R(0)\rangle + \langle R\rangle^2$, we obtain

$$\begin{aligned}\mathcal{L}[\langle \delta z(t)\delta z(0)\rangle_{ss}] &= \frac{\langle \delta z^2\rangle_{ss}}{s+\gamma} + \frac{1}{s^2-\gamma^2}\left[\int_0^\infty dt e^{-\gamma t}\langle \delta R(t)\delta R(0)\rangle - \int_0^\infty dt e^{-st}\langle \delta R(t)\delta R(0)\rangle\right] \\ &= \frac{\langle \delta z^2\rangle_{ss}}{s+\gamma} + \frac{\langle \delta R\rangle^2}{s^2-\gamma^2}\left[\hat{\phi}_R(\gamma) - \hat{\phi}_R(s)\right],\end{aligned} \tag{S1-21}$$

with $\hat{\phi}_q(s) = \int_0^\infty dt e^{-st}\,\langle \delta q(t)\delta q(0)\rangle / \langle \delta q^2\rangle$. By dividing both sides of Eq S1-21 by $\langle \delta z^2\rangle_{ss}$, we obtain

$$\hat{\phi}_z(s) = \frac{1}{s+\gamma} + \frac{\eta_R^2}{\eta_z^2}\frac{\gamma^2}{s^2-\gamma^2}\left[\hat{\phi}_R(\gamma) - \hat{\phi}_R(s)\right], \tag{S1-22}$$

where η_z^2 and η_R^2 are, respectively, the relative variance of the product number, $\eta_z^2 = \langle \delta z^2\rangle / \langle z\rangle^2$ and its rate, $\eta_R^2 = \langle \delta R^2\rangle / \langle R\rangle^2$. This result has been previously reported in Eq (D5) in ref.[2].

Noting that the Laplace transform of $\langle z(t)z(0)\rangle_{ss}$, or $\hat{\varphi}_z(s)$, is given by $\langle \delta z^2\rangle \hat{\phi}_z(s)$, we can obtain the expression for the power spectrum of the product number from Eq S1-3 and Eq S1-22 as follows:

$$S_z(\omega) = \langle \delta z^2 \rangle [\hat{\phi}_z(i\omega) + \hat{\phi}_z(-i\omega)]$$
$$= \frac{2\langle \delta z^2 \rangle \gamma}{\omega^2 + \gamma^2} \left[1 - \frac{\eta_R^2}{\eta_z^2} \gamma \hat{\phi}_R(\gamma) \right] + \frac{1}{\omega^2 + \gamma^2} \frac{\langle z \rangle^2}{\langle R \rangle^2} S_R(\omega). \tag{S1-23}$$

Here, $S_R(\omega)$ denotes the power spectrum of product creation rate, i.e., $S_R(\omega) = \langle \delta R^2 \rangle \left[\hat{\phi}_R(i\omega) - \hat{\phi}_R(-i\omega) \right]$. Noting that $\langle \delta z^2 \rangle \gamma^2 \, \eta_R^2 / \eta_z^2 = \langle z \rangle^2 \gamma^2 \eta_R^2 = \langle R \rangle^2 \eta_R^2 = \langle \delta R^2 \rangle$ and $\langle z \rangle^2 = \left(\langle R \rangle / \gamma \right)^2$, we can simplify Eq S1-23 to

$$S_z(\omega) = \frac{2}{\omega^2 + \gamma^2} \left[\gamma \langle \delta z^2 \rangle - \langle \delta R^2 \rangle \hat{\phi}_R(\gamma) \right] + \frac{S_R(\omega)}{\omega^2 + \gamma^2} \quad . \tag{S1-24}$$

It is known that, for the general birth-death process, the Fano factor, $F_z (\equiv \langle \delta z^2 \rangle / \langle z \rangle = \eta_z^2 \langle z \rangle)$, of the product noise obeys the following equation [2]:

$$F_z - 1 = \hat{\phi}_R(\gamma) F_R \tag{S1-25}$$

By multiplying $\langle z \rangle \left(= \langle R \rangle / \gamma \right)$ on both sides of Eq S1-25, we obtain $\langle \delta z^2 \rangle = \langle z \rangle + \hat{\phi}_R(\gamma) \langle \delta R^2 \rangle / \gamma$. Substituting this equation into Eq S1-24, we finally obtain

$$S_z(\omega) = \frac{2\langle R \rangle}{\omega^2 + \gamma^2} + \frac{S_R(\omega)}{\omega^2 + \gamma^2}, \tag{S1-26}$$

which is Eq 3 in the main text.

References

1. Sung J, Silbey RJ. Counting statistics of single molecule reaction events and reaction dynamics of a single molecule. Chem Phys Lett. 2005;415(1):10-4.

2. Lim YR, Kim J-H, Park SJ, Yang G-S, Song S, Chang S-K, et al. Quantitative Understanding of Probabilistic Behavior of Living Cells Operated by Vibrant Intracellular Networks. Phys Rev X. 2015;5(3):031014.
