# Supplementary material for: Frequency spectrum of chemical fluctuation: A probe of reaction mechanism and dynamics
Source: PLoS Comput Biol. 2019 Sep 16;15(9):e1007356. doi: 10.1371/journal.pcbi.1007356 (PMC6762214; doi:10.1371/journal.pcbi.1007356)
Supplement: S2 Text — (PDF) [file pcbi.1007356.s002.pdf]

Supplementary Text 2 | Power spectrum of the product creation rate for multi-channel and multi-step processes.

In this section, we briefly derive the power spectrum of the product creation rate for the multi-channel reaction process and the multi-step process, shown in Fig 1B and 1C. from the main text. For the *l*-channel process shown in Fig 1B, the time correlation function of the product creation rate can be written as

$$\langle R(t)R(0)\rangle_{ss} = \sum_{i=1}^{l}\sum_{j=1}^{l} R_j G(\Gamma_j, t \mid \Gamma_i) R_i P_{ss}(\Gamma_i) \tag{S2-1}$$

which is equivalent to Eq S1-19 for the case where the state variable is discrete. In Eq S2-1, R_i denotes the product creation rate when the reaction system is at state Γ_i. $G(\Gamma_j, t \mid \Gamma_i)$ denotes the propagator, or the conditional probability that the reaction system is at state Γ_j at time *t*, given that the system is at Γ_i at time 0. $P_{ss}(\Gamma_j)$ is the probability of finding the system at Γ_j in the steady state. In the long time limit, $G(\Gamma_j, t \mid \Gamma_i)$ should approach $P_{ss}(\Gamma_j)$ so that we have $\lim_{t\to\infty}\langle R(t)R(0)\rangle_{ss} = \langle R\rangle^2$. By subtracting $\langle R\rangle^2$ from both sides of Eq S2-1, we obtain

$$\langle \delta R(t)\delta R(0)\rangle_{ss} = \sum_{i=1}^{l}\sum_{j=1}^{l} R_j \left[G(\Gamma_j, t \mid \Gamma_i) - P_{ss}(\Gamma_j) \right] R_i P_{ss}(\Gamma_i)\,. \tag{S2-2}$$

For the multi-channel process, the propagator, $G(\Gamma_j, t \mid \Gamma_i)$, is given by the *j*-th element of the *l*-dimensional column vector $\mathbf{G}(t)$ that satisfies the following time-evolution equation

$$\frac{\partial}{\partial t}\mathbf{G}(t) = \mathbf{T}\cdot\mathbf{G}(t) \tag{S2-3}$$

and the following initial condition, $\left[\mathbf{G}(0)\right]_j = \delta_{ji}$. In Eq S2-3, $\mathbf{T}$ represents the transition rate matrix corresponding to the multi-channel reaction, that is,

$$\mathbf{T} = \begin{pmatrix} -k_{21} & k_{12} & 0 & 0 & \cdots \\ k_{21} & -(k_{12}+k_{32}) & k_{23} & 0 & \cdots \\ 0 & k_{32} & -(k_{23}+k_{43}) & k_{34} & \cdots \\ 0 & 0 & k_{43} & -(k_{34}+k_{54}) & \cdots \\ \vdots & \vdots & \vdots & \vdots & \ddots \end{pmatrix}$$

The solution of Eq S2-3 can be obtained as

$$G(\Gamma_j, t \mid \Gamma_i) = P_{ss}(\Gamma_j) + \sum_{k=2}^{l} (\mathbf{Q})_{jk} e^{-\lambda_k t} (\mathbf{Q}^{-1})_{ki}, \tag{S2-4}$$

where $\mathbf{Q}$ and $\{\lambda_k\}$ denote the matrix that diagonalizes $\mathbf{T}$, i.e., $\mathbf{Q}^{-1} \cdot \mathbf{T} \cdot \mathbf{Q} = \mathbf{\Lambda}$ with $(\mathbf{\Lambda})_{ij} = \lambda_i \delta_{ij}$, and the non-negative eigenvalues of the transition rate matrix. On the R.H.S. of Eq S2-4, $P_{ss}(\Gamma_j)$ corresponds to the term with zero eigenvalue, $\lambda_1 = 0$. Substituting Eq S2-4 into Eq S2-2, we obtain the following multi-exponential function for the TCF of the product creation rate:

$$\langle \delta R(t) \delta R(0) \rangle_{ss} = \langle \delta R^2 \rangle \sum_{k=2}^{l} c_k \exp(-\lambda_k \mid t \mid), \tag{S2-5}$$

where $\langle \delta R^2 \rangle c_k$ is defined by $\sum_{k=2}^{l} \sum_{i=1}^{l} \sum_{j=1}^{l} R_j (\mathbf{Q})_{jk} (\mathbf{Q}^{-1})_{ki} R_i p_{ss}(\Gamma_i)$. Here, c_k satisfies the normalization condition: $\sum_{k=2}^{l} c_k = 1$.

The power spectrum of the product creation rate can be calculated by taking the Fourier transform of Eq S2-5:

$$S_R(\omega) = 2\langle \delta R^2 \rangle \sum_{k=2}^{l} c_k \frac{\lambda_k}{\omega^2 + \lambda_k^2}. \tag{S2-6}$$

The simplest multi-channel process is the two-channel process in which $R_1 = k$ and $R_2 = 0$. For this simple case, the time correlation function of the reaction rate, given in Eq S2-5, becomes the simple exponential function,

$$\langle \delta R(t) \delta R(0) \rangle_{ss} = \langle \delta R^2 \rangle \exp(-\lambda t) \qquad \text{(S2-7)}$$

with $\lambda = k_{12} + k_{21}$. Here, k_{ij} denotes the rate of transition from state Γ_i to state Γ_j. For the two-state model, $\langle \delta R^2 \rangle$ is given by $k^2 k_{12} k_{21} / (k_{12} + k_{21})^2$. The power spectrum of the product creation rate for the two-channel process is given by

$$S_R(\omega) = 2 \langle \delta R^2 \rangle \frac{\lambda}{\omega^2 + \lambda^2}. \qquad \text{(S2-8)}$$

By substituting Eq S2-8 into Eq 3 in the main text, we obtain the power spectrum of product number for the two-channel process:

$$S_z(\omega) = \frac{2\langle R \rangle}{\omega^2 + \gamma^2} \left(1 + \frac{\lambda}{\omega^2 + \lambda^2} F_R \right) \qquad \text{(S2-9)}$$

Equations S2-7-S2-9 are used to calculate the theoretical results for the multi-channel process in Fig 1E-1G. The TCF of the product number for the multi-channel process, shown in Fig 1D, can be calculated by the inverse Fourier transform of Eq S2-9, whose analytic expression is suppressed here.

Next, we present the derivation of the power spectrum of the product creation rate for the multi-step process shown in Fig 1C. The multi-step product creation process shown Fig 1C is an example of a renewal process. The analytic expression for the reaction waiting time distribution of the multi-step product creation process is simple in the Laplace domain and given by [1, 2]

$$\hat{\psi}(s) = \prod_{j=1}^{n} \frac{k_j}{s + k_j} \quad , \tag{S2-10}$$

where n and k_j denote the number of intermediate reaction steps composing the product creation process and the rate of the j-th reaction step, respectively. By substituting Eq S2-10 into Eq 6, we can obtain the analytic expression for the power spectrum of the product creation rate for the multi-step reaction process. If we assume that all rates of the internal steps are the same, i.e., $k_l = k$, we can write the rate power spectrum as

$$S_R(\omega) = \frac{2k}{n} \mathrm{Re}\left\{ \frac{1}{[1 + i(\omega/k)]^n - 1} \right\} \quad , \tag{S2-11}$$

where we exploit the fact that $\langle R \rangle = k/n$. Eq S2-11 then can be rewritten as

$$S_R(\omega) = \frac{2k}{n} \left[\frac{[1 + (\omega/k)^2]^{n/2} \cos n\theta - 1}{[1 + (\omega/k)^2]^n - 2[1 + (\omega/k)^2]^{n/2} + 1} \right] \quad , \tag{S2-12}$$

where θ is defined as $\theta = \tan^{-1}(\omega/k)$. Equation S2-12 and its inverse Fourier transform are used to calculate the power spectrum and the TCF of the product creation rate, respectively, in Fig 1G and 1F, for the multi-step process. Substituting Eq S2-12 into Eq 3 in the main text, we can obtain the power-spectrum of the product number for the multi-step reaction, and the associated TCF of the product number fluctuation by calculating its inverse Fourier transform, which are shown as the blue lines in Fig 1E and D, respectively.

References

1. Cox DR. Renewal theory. 1962.

2. Jung W, Yang S, Sung J. Novel chemical kinetics for a single enzyme reaction: relationship between substrate concentration and the second moment of enzyme reaction time. J Phys Chem B. 2010;114(30):9840-7.
