# Supplementary material for: Frequency spectrum of chemical fluctuation: A probe of reaction mechanism and dynamics
Source: PLoS Comput Biol. 2019 Sep 16;15(9):e1007356. doi: 10.1371/journal.pcbi.1007356 (PMC6762214; doi:10.1371/journal.pcbi.1007356)
Supplement: S3 Text — (PDF) [file pcbi.1007356.s003.pdf]

Supplementary Text 3 | Simulation method for Fig 1.

Here, we provide the algorithms used to simulate the three different creation reactions shown in Fig 1A-1C. We also discuss how to calculate the time-correlation function of the product number, $\langle \delta z(t)\delta z(0)\rangle$, and the power spectrum of the product number, $S_z(\omega)$, from the simulation results.

A. Simple Poisson birth-death process

When product creation is a Poisson process, its dynamics can be completely characterized by a single rate constant, *R*; the reaction waiting time distribution of the Poisson product creation process is given by $\varphi_r(t) = R e^{-Rt}$. The reaction waiting time, t_i, of the *i*-th Poisson product creation event can be generated by $t_i = -R^{-1} \ln u_i$ with u_i being the uniformly distributed random variable between 0 and 1. In Fig 1, we set the value of *R* equal to 2 in an arbitrary time unit.

Throughout this work, the product degradation process is assumed to a Poisson process. When each product molecule is generated in the simulation, we also generate the lifetime of each product molecule from the degradation waiting time distribution, $\varphi_d(t) = \gamma e^{-\gamma t}$, where the γ is a product's degradation rate constant. That is to say, the lifetime of the *i*-th product molecule is determined from another uniformly distributed random variable, u_i' between 0 and 1 by $\tau_i = -\gamma^{-1} \ln u_i'$. In Fig 1, we set the value of γ equal to 1.

Performing this simulation iteratively, we can generate the number of the time traces of the product number. By taking the average over the time traces of the product number, we obtain the time-dependent mean and variance of the product number. The mean and variance

in the product number reach the steady-state values at long times. In our simulation, the steady-state is found to be attained at times longer than $5\gamma^{-1}$.

We calculate the steady-state time autocorrelation function of the product number from the simulation time traces of the product number as follows. We first set the value of t_0 as $5\gamma^{-1}$. The mean product number at time t_0 is essentially the same as the steady-state value, $\langle z\rangle_{ss}$. For each trajectory, we calculate $\delta z(t_0)$ and $\delta z(t+t_0)$, where $\delta z(t)$ designates $z(t)-\langle z\rangle_{ss}$. By performing the average of $\delta z(t+t_0)\delta z(t_0)$ over the simulation trajectories, we obtain the value of the steady-state TCF, $\langle\delta z(t+t_0)\delta z(t_0)\rangle_{ss}$, of the product number. We confirm that the value of $\langle\delta z(t+t_0)\delta z(t_0)\rangle_{ss}$ is independent of t_0, as long as the value of t_0 is greater than $5\gamma^{-1}$.

To obtain the rate time autocorrelation function, $\langle\delta R(t)\delta R(0)\rangle$ (Fig 1F), we perform the simulation with synchronized initial condition, i.e. all independent realizations of the reaction begin at the time 0. Then, we calculate the mean number, $\langle n(t)\rangle^{*}$, where asterisk denotes the synchronized initial condition. According to the ref. [1], we obtain the $\langle\delta R(t)\delta R(0)\rangle$ using $\langle R\rangle d\langle n(t)\rangle^{*}/dt-\langle R\rangle^{2}$.

From the simulated time traces of the product number at times longer than t_0, the power spectrum of the product number is obtained from a discrete Fourier transform of the deviation of the product number fluctuation, according the definition of the power spectrum, given in Eq 1 in the main text.

B. Multi-channel creation process with constant rate decay

In this section, we describe the algorithm used to simulate the multi-channel creation reaction shown in Fig 1B. As shown in Fig 1B, the reaction state fluctuates with the state-traversing rate, k_{ij}, which denotes the state-transition rate constant from the Γ_i-state to the Γ_j-state. Each state, Γ_i, has its own product creation rate constant, R_i.

We start the simulation by sampling the initial state using the steady-state state distribution. On sampling the initial state, Γ_i, we generate the state-transition waiting time by using the transition rate constant from the Γ_i state to the adjacent state. If there are two transition directions, we generate the transition waiting time for each direction and choose the shorter one. Once we sample the transition waiting time, we then conduct a simple Poisson product creation reaction simulation by using the state's creation rate constant, R_i, until the time reaches the state-transition waiting time. After each product creation process ends, we calculate the product lifetime using the same procedure described in previous section. When the time reaches the previously sampled state-transition time, we change the creation reaction rate constant into the new rate constant, R_j, for the new state, Γ_j. We then repeatedly conduct the sampling of the state-transition waiting time and a simple Poisson product creation simulation using the new rate for the sampled state transition waiting time.

By continually repeating this procedure, we can simulate the multi-channel creation process. Finally, we calculate the time correlation and the power spectrum of the product number fluctuation in the same way as the simple Poisson creation case. For the result of Fig 1D and 1E, we choose the simplest multi-channel reaction process, two-state reaction process. When we denote the two-states as the on-state and the off-state, we set the state transition rate from each of the two states as the same as 1/4 in the simulation. The product creation reaction

occurs only when our system is at the on-state, and the product creation rate is set to be 4 in our simulation. We set the rate of the product decay process, γ, as 1.

C. Multi-step creation process with constant rate decay

To simulate the multi-step reaction process shown in Fig 1C, we generate the creation reaction time by summing the set of the reaction waiting times of the Poisson reaction steps composing the multi-step reaction process. The other details of the simulation method are the same as the other cases described in the previous sections.

In this simulation, we set the number of the intermediate steps composing a single reaction equal to 20 and, for each step, the catalytic rate in each step to 40, in order to set the mean product creation time to 1/2, the same as the mean product creation time set in the simulation of other reaction schemes shown in Fig 1. The decay process rate constant, γ, is set equal to 1, as well.

At long times, the simulated dynamics of the product number statistics reaches the steady-state. However, one can obtain the steady-state product number trajectories from the beginning of the simulation by sampling the first reaction waiting time according to

$$\psi_1(t) = S(t) \Big/ \int_0^\infty dt' S(t'), \qquad \text{(S3-1)}$$

where $S(t)$ denotes the probability that the multi-step reaction has not yet completed as of time *t*, given that the multi-step reaction started at time 0. Alternatively, we can start our simulation at one of the intermediate reaction steps. The initial sampling probability of the *i*-th intermediate reaction step is given by $\tau_i \Big/ \left(\sum_{j=1}^{n} \tau_j \right)$ with τ_i being the mean lifetime of the intermediate step, i.e., $\tau_i = k_i^{-1}$.

Reference

1. Park SJ, Song S, Yang G-S, Kim PM, Yoon S, Kim J-H, et al. The Chemical Fluctuation Theorem governing gene expression. Nat Commun. 2018;9(1):297.
