# Supplementary material for: Frequency spectrum of chemical fluctuation: A probe of reaction mechanism and dynamics
Source: PLoS Comput Biol. 2019 Sep 16;15(9):e1007356. doi: 10.1371/journal.pcbi.1007356 (PMC6762214; doi:10.1371/journal.pcbi.1007356)
Supplement: S4 Text — (PDF) [file pcbi.1007356.s004.pdf]

Supplementary Text 4 | Derivation of Eq 6.

In this Method, we will begin by deriving the exact relationship between the time correlation function (TCF) of the product creation rate and the time series of reaction events, or the series of times at which reaction event occurs. In the ref. [1], the Laplace domain expression for normalized rate autocorrelation is given by

$$F_R\hat{\phi}_R(s) = \sum_{l=0}^{\infty}\hat{\psi}_l(s) - \frac{\langle R\rangle}{s} \quad , \tag{S4-1}$$

where F_R is the Fano factor of the creation rate, $F_R = \langle \delta R^2\rangle / \langle R\rangle$ and $\psi_l(t)$ denotes the lth reaction time distribution. Substituting Eq S4-1 into Eq S1-3, or $S_R(\omega) = 2\langle R\rangle F_R \lim_{\varepsilon\to 0^+} \mathrm{Re}\left[\hat{\phi}_R(\varepsilon + i\omega)\right]$, we can easily obtain the expression for the power spectrum of the product creation rate.

For the special case when the product creation process in a renewal process, we have $\hat{\psi}_l(s) = \hat{\psi}_1(s)^l$, so that Eq S4-1 becomes

$$F_R\hat{\phi}_R(s) = \frac{\hat{\psi}_1(s)}{1-\hat{\psi}_1(s)} - \frac{\langle R\rangle}{s} \quad . \tag{S4-2}$$

Substituting Eq S4-1 into Eq S1-3, or $S_R(\omega) = 2\langle R\rangle F_R \lim_{\varepsilon\to 0^+} \mathrm{Re}\left[\hat{\phi}_R(\varepsilon + i\omega)\right]$, we obtain the following expression for the power spectrum, $S_R(\omega)$, of the product creation rate for a renewal product creation process:

$$S_R(\omega) = 2\langle R\rangle \,\mathrm{Re}\left[\frac{\hat{\psi}_1(i\omega)}{1-\hat{\psi}_1(i\omega)}\right] - 2\langle R\rangle^2 \lim_{\varepsilon\to 0^+}\frac{\varepsilon}{\varepsilon^2+\omega^2} \tag{S4-3}$$

Noticing that $\psi_1(t)$ is zero when $t<0$, we define the Fourier transform of $\psi_1(t)$ as $\tilde{\psi}_1(\omega)=\int_0^\infty dte^{-i\omega t}\psi_1(t)$. Then, the above equation can also be written as

$$S_R(\omega)=2\langle R\rangle \mathrm{Re}\left[\frac{\tilde{\psi}(\omega)}{1-\tilde{\psi}(\omega)}\right]-2\pi\langle R\rangle^2\delta(\omega) \qquad \text{(S4-4)}$$

For nonzero frequency $(\omega>0)$, Eq S4-4 is the same as Eq 5 in the main text.

Reference

1. Park SJ, Song S, Yang G-S, Kim PM, Yoon S, Kim J-H, et al. The Chemical Fluctuation Theorem governing gene expression. Nat Commun. 2018;9(1):297.
