# Supplementary material for: Frequency spectrum of chemical fluctuation: A probe of reaction mechanism and dynamics
Source: PLoS Comput Biol. 2019 Sep 16;15(9):e1007356. doi: 10.1371/journal.pcbi.1007356 (PMC6762214; doi:10.1371/journal.pcbi.1007356)
Supplement: S5 Text — (PDF) [file pcbi.1007356.s005.pdf]

Supplementary Text 5 | Analytic expressions for power spectra in Fig 3.

In this section, we discuss the power spectra visualized in Fig 3. According to Eq 3, the protein number power spectrum is related with the power spectrum of the translation rate by

$$S_p(\omega) = S_p^0(\omega) + \frac{S_{R_{TL}}(\omega)}{\omega^2 + \gamma_p^2}, \tag{S5-1}$$

where $S_p^0(\omega) = 2\langle R_{TL}\rangle / (\omega^2 + \gamma_p^2)$. Because $R_{TL} = mk_{TL}$ for our model, the above equation can be expressed as

$$S_p(\omega) = S_p^0(\omega) + \frac{k_{TL}^2 S_m(\omega)}{\omega^2 + \gamma_p^2}, \tag{S5-2}$$

where $S_m(\omega)$ is the mRNA number power spectrum. Using the Eqs S5-1 and S5-2, we can find the form of $S_p(\omega)/S_p^0(\omega) - 1$ as

$$\frac{S_p(\omega)}{S_p^0(\omega)} - 1 = \frac{k_{TL} S_m(\omega)}{2\langle m\rangle}, \tag{S5-3}$$

where $\langle m\rangle$ is the mean number of mRNA. To obtain the analytic expression of $S_m(\omega)$, we use Eq 3 again. The result is given by

$$S_m(\omega) = \frac{2\langle R_{TX}\rangle}{\omega^2 + \gamma_m^2} + \frac{S_{R_{TX}}(\omega)}{\omega^2 + \gamma_m^2}. \tag{S5-4}$$

Here, the transcription rate is given by $R_{TX} = k_{TX}\xi$, where the value of ξ takes 1 for the active gene state but 0 for the inactive gene state. Using this expression of the translation rate, we obtain $S_{R_{TX}}(\omega)$ as $S_{R_{TX}}(\omega) = k_{TX}^2 S_\xi(\omega)$.

The analytic expression of $S_\xi(\omega)$ can be obtained from the Fourier transform of the time correlation function of the gene state variable ξ. According to ref [1], the time correlation function of the gene state variable in our model is given in the Laplace domain by

$$\mathcal{L}[\langle \delta\xi(t)\delta\xi(0)\rangle] \equiv \langle \delta\xi^2\rangle \hat{\phi}_\xi(s) = \frac{\tau_{on}\tau_{off}}{(\tau_{on}+\tau_{off})^2}\frac{1}{s} - \frac{1}{s^2(\tau_{on}+\tau_{off})}\frac{[1-\hat{\psi}_{on}(s)][1-\hat{\psi}_{off}(s)]}{1-\hat{\psi}_{on}(s)\hat{\psi}_{off}(s)} \ , \tag{S5-5}$$

where τ_{on} and τ_{off} are the first moments of waiting time distributions, ψ_{on} and ψ_{off}, respectively. The Fourier transform of this equation is given by

$$S_\xi(\omega) = \langle \delta\xi^2\rangle \lim_{\varepsilon\to 0^+}[\phi_\xi(i\omega+\varepsilon)+\phi_\xi(-i\omega+\varepsilon)] = \langle \delta\xi^2\rangle \tilde{\phi}_\xi(\omega). \tag{S5-6}$$

Substituting Eq S5-5 into Eq S5-6, we obtain

$$S_{R_{TX}}(\omega) = k_{TX}^2 S_\xi(\omega) = k_{TX}^2\langle \delta\xi^2\rangle \tilde{\phi}_\xi(\omega) = \frac{\tau_{on}\tau_{off}}{(\tau_{on}+\tau_{off})^2}\pi\delta(\omega) + \frac{k_{TX}^2}{\tau_{on}+\tau_{off}}\frac{\tilde{G}(\omega)}{\omega^2}, \tag{S5-7}$$

where $\tilde{G}(\omega)$ is given by $\tilde{G}(\omega) = \lim_{\varepsilon\to 0^+} 2\,\mathrm{Re}[\hat{G}(\varepsilon+i\omega)]$ with

$$\hat{G}(s) \equiv \frac{[1-\hat{\psi}_{on}(s)][1-\hat{\psi}_{off}(s)]}{1-\hat{\psi}_{on}(s)\hat{\psi}_{off}(s)}. \tag{S5-8}$$

Substituting the second equality of Eq S5-7 into Eq S5-4, we obtain the power spectrum of the mRNA number by

$$S_m(\omega) = \frac{k_{TX}\langle\xi\rangle}{\omega^2+\gamma_m^2}\left[2 + k_{TX}\tilde{\phi}_\xi(\omega)F_\xi\right] \ , \tag{S5-9}$$

where $F_\xi = \langle \delta\xi^2\rangle / \langle\xi\rangle$. By substituting Eq S5-9 into S5-3, we obtain the power spectrum of the protein number as follows:

$$\frac{S_p(\omega)}{S_p^0(\omega)} - 1 = \frac{k_{TL}\gamma_m}{\omega^2 + \gamma_m^2}\left[1 + \frac{1}{2}k_{TX}\tilde{\phi}_\xi(\omega)F_\xi\right]. \tag{S5-10}$$

In Eqs S5-9 and S5-10, $\tilde{\phi}_\xi(\omega)F_\xi$ is given by

$$\tilde{\phi}_\xi(\omega)F_\xi = \frac{\tilde{G}(\omega)}{\tau_{on}\omega^2} \tag{S5-11}$$

for nonzero frequency. The definition of $\tilde{G}(\omega)$ can be found below Eq S5-7.

To obtain the high frequency asymptotic behavior of $S_p(\omega)/S_p^0(\omega) - 1$, we note that the large s limit value of $\hat{G}(s)$ given in Eq S5-8 is given by $\lim_{s\to\infty}\hat{G}(s) = 1$, because $\lim_{s\to\infty}\hat{\psi}_{on(off)}(s) = 0$. This means that $\tilde{G}(\omega)\left[\equiv 2\lim_{\varepsilon\to 0^+}\mathrm{Re}\,\hat{G}(i\omega+\varepsilon)\right]$ becomes 2 in the high frequency limit. Therefore, $\tilde{\phi}_\xi(\omega)F_\xi$ given in Eq S5-11 yields the following high frequency asymptotic behavior:

$$\frac{1}{2}\tilde{\phi}_\xi(\omega)F_\xi \xrightarrow{\omega\to\infty} \frac{1}{\tau_{on}\omega^2}. \tag{S5-12}$$

With Eq S5-12 at hand, we obtain the asymptotic behavior of $S_p(\omega)/S_p^0(\omega) - 1$ given in Eq S5-10 as follows:

$$\frac{S_p(\omega)}{S_p^0(\omega)} - 1 \xrightarrow{\omega\to\infty} \frac{k_{TL}\gamma_m}{\omega^2}. \tag{S5-13}$$

Reference

1. Goychuk I, Hänggi P. Non-Markovian stochastic resonance. Phys Rev Lett. 2003;91(7):070601.
