# Supplementary material for: Frequency spectrum of chemical fluctuation: A probe of reaction mechanism and dynamics
Source: PLoS Comput Biol. 2019 Sep 16;15(9):e1007356. doi: 10.1371/journal.pcbi.1007356 (PMC6762214; doi:10.1371/journal.pcbi.1007356)
Supplement: S6 Text — (PDF) [file pcbi.1007356.s006.pdf]

Supplementary Text 6 | Simulation method for Fig 3.

Here, we describe the method used to simulate the gene expression network shown in Fig 3. This simulation consists of the three states: the gene activity fluctuation, the transcription process, and the translation process.

A. Gene activity fluctuation

The first step in this simulation is to sample the initial state of the gene according the steady-state distribution. The steady-state probability of the active or inactive gene state is given by $p_{on} = k_{on}/(k_{on} + k_{off})$ or $p_{off} = 1 - p_{on}$, where k_{on}^{-1} and k_{off}^{-1} denote the average lifetimes of the inactive and active gene states, respectively, i.e., $k_{on}^{-1} = \int_0^t dt\psi_{off}(t)t\left(\equiv \tau_{off}\right)$ and $k_{off}^{-1} = \int_0^t dt\psi_{off}(t)t\left(\equiv \tau_{on}\right)$. If the uniform random number generated between 0 and 1 is less than p_{on}, we choose the active gene state; otherwise, we choose the inactive gene state. Given that the initial gene state is the active or inactive gene state, we generate the lifetime of the active gene state according to the lifetime distribution, $\psi_{on}(t)$ or $\psi_{off}(t)$. To simulate the gene activity fluctuation process, we repeatedly sample the lifetime of the active gene state and the lifetime of the inactive gene state one after the other.

B. Transcription process with activated gene

The second step we take is to simulate the transcription process. In the model shown in Fig 2A, the transcriptional rate is given by $R_{TX} = k_{TX}\xi$, where k_{TX} is the transcription rate constant for the gene in the active state. ξ is a stochastic variable whose value is either 1 for the active gene state and 0 for the inactive gene state. To simulate the transcription process, we simulate Poisson transcription events only when the gene state is in the active state. The rest of

the simulation method is the same as the simulation method for the multi-channel reaction described in S3 Text, Section B. In this way, we can generate the number of the time traces of the mRNA number. In this simulation, we take the value of k_{TX} as $k_{TX} = 0.51\ \mathrm{min}^{-1}$, which is given in reference [1].

From the simulation trajectories of the mRNA number, we can calculate various measures of the product number counting statistics, such as the mean, the variance, the time correlation function, and the distribution of the mRNA number, as described in S3 Text.

C. Translation process with live mRNA

The last simulation step is the translation process. In the model shown in Fig 2A, the translation rate is given by $k_{TL}m$. This means that, for each mRNA, proteins are created by the translation rate, k_{TL}. To simulate this model, for each mRNA, we simulate the Poisson translation process, or the protein creation process, with the rate k_{TL} during the lifetime of the mRNA, using the same method as that described in S4 Text, Section A. Because the mRNA lifetime distribution is an exponential function in our model, upon each creation of mRNA, we generate the lifetime of the mRNA by $\tau_i = -\gamma_m^{-1} \ln u_i$, where u_i is a uniformly distributed random variable between 0 and 1. Likewise, upon each generation of protein molecule, the exponentially distributed lifetime of the protein molecule with the mean lifetime γ_p^{-1} is generated in the same manner, and this information is then used in generating the simulation trajectories of the protein number. From the simulation trajectories of protein number, we can calculate the power-spectrum and the time-correlation function of the protein number, shown in Fig 2, using the method described above. In this simulation, we take the values of k_{TX} and γ_p as $k_{TX} = 1.695\ \mathrm{min}^{-1}$ and $\gamma_p = 3.18 \times 10^{-2}\ \mathrm{min}^{-1}$, respectively [1].

In the calculation of the power spectrum of the mRNA or the protein number from the simulation trajectories, we perform the discrete Fourier transform with the Kaiser window function [2], which is a commonly used window function for random signal analysis. The Kaiser window function is given by

$$W(n) = \frac{I_0\left(\pi\alpha\sqrt{1-(2n/(N-1)-1)^2}\right)}{I_0(\pi\alpha)}, \qquad \text{(S6-1)}$$

where $I_0(x)$ denotes a modified Bessel function of order 0. In Eq S6-1, α and N denote the flattening level and the number of data in each time trajectory, respectively. In our work, the values of α and N are set equal to 3 and 50000, respectively.

References

1. Zoller B, Nicolas D, Molina N, Naef F. Structure of silent transcription intervals and noise characteristics of mammalian genes. Mol Syst Biol. 2015;11(7):823.
2. Kuo FF, Kaiser JF. System analysis by digital computer: Wiley; 1966.
