# Supplementary material for: Frequency spectrum of chemical fluctuation: A probe of reaction mechanism and dynamics
Source: PLoS Comput Biol. 2019 Sep 16;15(9):e1007356. doi: 10.1371/journal.pcbi.1007356 (PMC6762214; doi:10.1371/journal.pcbi.1007356)
Supplement: S7 Text — (PDF) [file pcbi.1007356.s007.pdf]

Supplementary Text 7 | Power spectrum analysis for approximated $\psi_{off}(t)$.

In this method, we present the details of the power-spectrum analysis for approximated $\psi_{off}(t)$. The gene-expression network model used in the analysis is the same as Fig 2A. However, instead of assuming the particular gene activation dynamics assumed in Fig 2B, we use a more general but simpler model of the gene activation process, in which the reaction waiting time distribution of the gene activation, or the waiting time distribution of the inactive gene state, is given by a gamma distribution, $\psi_{off}(t) = t^{a-1}\exp\left[-t/b\right] / \left(\Gamma(a)b^a\right)$. In this analysis, we assume $\psi_{on}(t)$ as an exponential distribution, i.e. $\psi_{on}(t) = k_a \exp[-k_a t]$, where k_a is the rate of the gene-deactivation process. For this model, the Laplace transform of $\psi_{on}(t)$ and $\psi_{off}(t)$ are given by $\hat{\psi}_{on}(s) = (s+k_a)^{-1}$ and $\hat{\psi}_{off}(s) = (1+sb)^{-a}$, respectively. Substituting these equations into Eq S5-7, we can obtain the fully explicit analytic expression of $S_{R_{TX}}(\omega)$ as

$$S_{R_{TX}}(\omega) = \frac{2k_{TX}^2\tau_{on}^2}{\tau_{on}+\tau_{off}} \frac{1-\dfrac{\cos[a\tan^{-1}(b\omega)]}{(1+b^2\omega^2)^{a/2}}}{\left(1-\dfrac{\cos[a\tan^{-1}(b\omega)]}{(1+b^2\omega^2)^{a/2}}\right)^2 + \left(\tau_{on}\omega + \dfrac{\sin[a\tan^{-1}(b\omega)]}{(1+b^2\omega^2)^{a/2}}\right)^2} . \qquad \text{(S7-1)}$$

where τ_{off} is equal to ab. This equation is compared with the data for $S_{R_{TX}}(\omega)$, which can be obtained from the data for the protein number power spectrum, $S_p(\omega)$, by repeated use of Eqs 8 and 9.

Alternatively, we can directly analyze the protein number power spectrum data. By substituting Eq S7-1 into Eq S5-4, we obtain the power-spectrum, $S_m(\omega)$, of the mRNA number. Then, substituting this equation into Eq S5-3, we obtain the following expression of $S_p(\omega) / S_p^0(\omega) - 1$:

$$\frac{S_p(\omega)}{S_p^0(\omega)}-1=\frac{k_{TL}\gamma_m}{\omega^2+\gamma_m^2}\left[1+k_{TX}\tau_{on}\frac{1-\frac{\cos[a\tan^{-1}(b\omega)]}{(1+b^2\omega^2)^{a/2}}}{\left(1-\frac{\cos[a\tan^{-1}(b\omega)]}{(1+b^2\omega^2)^{a/2}}\right)^2+\left(\tau_{on}\omega+\frac{\sin[a\tan^{-1}(b\omega)]}{(1+b^2\omega^2)^{a/2}}\right)^2}\right]. \quad \text{(S7-2)}$$

This equation can be compared with the data for the protein number power spectrum, $S_p(\omega)$.

In Eqs S7-1 and S7-2, the adjustable parameters are a, b, and . This is because τ_{off} is the same as ab, and k_{TX} is related to the mean transcription rate $\langle R_{TL}\rangle$ by the following equation:

$$\frac{k_{TX}\tau_{on}}{\tau_{on}+\tau_{off}}=\langle R_{TX}\rangle=\gamma_m\langle m\rangle. \quad \text{(S7-3)}$$

The mean mRNA number can be directly estimated from the mean protein number, $\langle p\rangle$, from $\langle m\rangle=\langle p\rangle/k_{TL}$, where the value of k_{TL} can be, in turn, estimated from the high frequency asymptotic behavior of $S_p(\omega)$ given in Eq S5-13. Throughout this work, we assume that the values of γ_m, γ_p, and $\langle p\rangle$ are provided from the experimental data.

We find that this result provides a good quantitative explanation of the power-spectra of the transcription rate shown in Fig 3F, which are obtained from a simulation of the transcription network with $\hat{\psi}_{off}(s)=\hat{f}(\mathbf{k},s)$. $\psi_{on}(t)$ and $\psi_{off}(t)$ are optimized from the quantitative analysis of $S_{R_{TX}}(\omega)$ with use of $k_a'e^{-k_a't}$ and $\psi_{off}(t)=f'(a,b,t)$, and as can be seen in Fig 4A and 4B, optimized $\psi_{on}(t)$ and $\psi_{off}(t)$ are in good agreement with the original model using $\psi_{on}(t)=k_ae^{-k_at}$ and $\psi_{off}(t)=f(\mathbf{k},t)$.

It is worth mentioning that, when the rates of the n consecutive reaction processes composing the gene activation process are the same, i.e., when $k_1 = k_2 = \cdots = k_n = k$, the gamma distribution, $f'(n, k^{-1}, t)$, is the exact lifetime distribution, $\psi_{off}(t)\left[= f(\mathbf{k}, t)\right]$, of the inactive gene state. For the transcription network model shown in Fig 3A, the values of the seven rate parameters in the multi-step gene activation process do not greatly differ from each other. Therefore, the success of our analysis, using the gamma distribution as an approximate representation of $\psi_{off}(t)$, is not surprising.
