# Supplementary material for: Frequency spectrum of chemical fluctuation: A probe of reaction mechanism and dynamics
Source: PLoS Comput Biol. 2019 Sep 16;15(9):e1007356. doi: 10.1371/journal.pcbi.1007356 (PMC6762214; doi:10.1371/journal.pcbi.1007356)
Supplement: S8 Text — (PDF) [file pcbi.1007356.s008.pdf]

Supplementary Text 8 | Analytic expressions for power spectrum of the transcription rate in Fig 4.

In this method, we present the mathematical details related to the power spectrum analysis given in Fig 4. The translational part in the gene-expression network model used in Fig 4 is the same as in Fig 2A. However, the transcriptional part is here extended to a more general case in which the active gene transcription rate, k_{TX}, is treated as a dynamic stochastic variable. The mean value, $\langle k_{TX}\rangle$, of the active gene transcription rate is the same as the value of k_{TX} in Figs 3 and 4. The normalized time correlation function of k_{TX} is given by an exponentially decaying function of time, i.e., $\phi_{k_{TX}}(t)=\exp(-\lambda t)$. The gene regulation by the promoter is modelled to be the same as Model II in Fig S2B. The mean-scaled time correlation function of the total transcription rate, $R_{TX}(=k_{TX}\xi)$, can be written as [1]

$$\frac{\langle \delta R_{TX}(t)\delta R_{TX}(0)\rangle}{\langle R_{TX}\rangle^2}=\eta_{k_{TX}}^2\phi_{k_{TX}}(t)+\eta_\xi^2\phi_\xi(t)+\eta_{k_{TX}}^2\eta_\xi^2\phi_{k_{TX}}(t)\phi_\xi(t) \tag{S8-1}$$

unless k_{TX} is correlated with ξ. The Fourier transform of Eq S8-1 is given by

$$\frac{S_{R_{TX}}(\omega)}{\langle R_{TX}\rangle^2}=\eta_{k_{TX}}^2\tilde{\phi}_{k_{TX}}(\omega)+\eta_\xi^2\tilde{\phi}_\xi(\omega)+\eta_{k_{TX}}^2\eta_\xi^2\tilde{\phi}_{k_{TX}}(\omega)*\tilde{\phi}_\xi(\omega) \tag{S8-2}$$

where $f(\omega)*g(\omega)$ denotes the convolution integral defined by $f(\omega)*g(\omega)\equiv\frac{1}{2\pi}\int_{-\infty}^{\infty}d\omega' f(\omega-\omega')g(\omega')$. Note here that the Fourier transform of $\phi_{k_{TX}}(t)\phi_\xi(t)$ can be calculated as

$$\begin{aligned}\int_{-\infty}^{\infty}dte^{-i\omega t}\phi_{k_{TX}}(t)\phi_\xi(t)&=\int_{-\infty}^{\infty}dte^{-i\omega t}\phi_{k_{TX}}(t)\left[\frac{1}{2\pi}\int_{-\infty}^{\infty}d\omega' e^{i\omega' t}\tilde{\phi}_\xi(\omega')\right]\\&=\frac{1}{2\pi}\int_{-\infty}^{\infty}d\omega'\tilde{\phi}_\xi(\omega')\int_{-\infty}^{\infty}dte^{-i(\omega-\omega')t}\phi_{k_{TX}}(t)\\&=\frac{1}{2\pi}\int_{-\infty}^{\infty}d\omega'\tilde{\phi}_\xi(\omega')\tilde{\phi}_{k_{TX}}(\omega-\omega')\end{aligned} \tag{S8-3}$$

In terms of the mean-scaled power spectrum, $\tilde{S}_q(\omega) \equiv S_q(\omega) \big/ \langle q \rangle^2$, Eq S8-2 can be rewritten as

$$\tilde{S}_{R_{TX}}(\omega) = \tilde{S}_{k_{TX}}(\omega) + \tilde{S}_{\xi}(\omega) + \tilde{S}_{k_{TX}}(\omega) * \tilde{S}_{\xi}(\omega) \tag{S8-4}$$

which is the same as Eq 11 in the main text. In the total transcription rate, $R_{TX}(= k_{TX}\xi)$, considered in Text S7, the active gene transcription rate, k_{TX}, is simply a constant. Therefore, dividing both sides of Eq S7-1 by k_{TX}^2, one can obtain the expression of $S_{\xi}(\omega)$. In case of $S_{k_{TX}}(\omega)$, its expression is simply given by Eq S2-8 with *R* being equal to k_{TX}. For convenience, the explicit expressions of $\tilde{S}_{\xi}(\omega)$ and $\tilde{S}_{k_{TX}}(\omega)$ are presented below:

$$\tilde{S}_{\xi}(\omega) = 2(\tau_{on} + \tau_{off}) \frac{1 - \dfrac{\cos[a \tan^{-1}(b\omega)]}{(1+b^2\omega^2)^{a/2}}}{\left(1 - \dfrac{\cos[a \tan^{-1}(b\omega)]}{(1+b^2\omega^2)^{a/2}}\right)^2 + \left(\tau_{on}\omega + \dfrac{\sin[a \tan^{-1}(b\omega)]}{(1+b^2\omega^2)^{a/2}}\right)^2} \,. \tag{S8-5}$$

$$\tilde{S}_{k_{TX}}(\omega) = 2\eta_{k_{TX}}^2 \frac{\lambda}{\omega^2 + \lambda^2} \,. \tag{S8-6}$$

Reference

1. Lim YR, Kim J-H, Park SJ, Yang G-S, Song S, Chang S-K, et al. Quantitative Understanding of Probabilistic Behavior of Living Cells Operated by Vibrant Intracellular Networks. Phys Rev X. 2015;5(3):031014.
