# Supplementary material for: Frequency spectrum of chemical fluctuation: A probe of reaction mechanism and dynamics
Source: PLoS Comput Biol. 2019 Sep 16;15(9):e1007356. doi: 10.1371/journal.pcbi.1007356 (PMC6762214; doi:10.1371/journal.pcbi.1007356)
Supplement: S9 Text — (PDF) [file pcbi.1007356.s009.pdf]

Supplementary Text 9 | Simulation method for Fig 4.

Here, we present a detailed description of the simulation method for the gene expression network model shown in Fig 4A. In this model, the transcriptional rate is given by $R_{TX} = k_{TX}(\Gamma)\xi$, where $k_{TX}(\Gamma)$ is the active gene transcription rate that is dependent on the cell state, Γ. ξ is a dichotomous stochastic variable whose value is either 1 for the active gene state and 0 for the inactive gene state. To simulate the stochastic fluctuation in k_{TX}, the normalized time correlation function of which is given by $\phi_{k_{TX}}(t) = \exp(-\lambda t)$, we adopt the two-state model, where k_{TX} dynamically fluctuates between $k_{TX}^{(1)}[\equiv k_{TX}(\Gamma_1)]$ and $k_{TX}^{(2)}[\equiv k_{TX}(\Gamma_2)]$ with transition rates k_{12} and k_{21}. Here, k_{ij} denotes the transition rate from Γ_j to Γ_i. For this model, the mean, noise, and inverse of relaxation time of k_{TX} is given by

$$\langle k_{TX} \rangle = k_{TX}^{(1)} \frac{k_{12}}{k_{12}+k_{21}} + k_{TX}^{(2)} \frac{k_{21}}{k_{12}+k_{21}} \quad \text{(S9-1)}$$

$$\eta_{k_{TX}}^2 = \frac{k_{12}k_{21}[k_{TX}^{(1)} - k_{TX}^{(2)}]^2}{[k_{TX}^{(1)}k_{12} + k_{TX}^{(2)}k_{21}]^2} \quad \text{(S9-2)}$$

$$\lambda = k_{12} + k_{21} \quad \text{(S9-3)}$$

Using the above equations, we can determine the values of the rate parameters that reproduces the given values of $\langle k_{TX} \rangle$, $\eta_{k_{TX}}^2$, and λ. For each mRNA time trace, initial values of k_{TX} and ξ are sampled with their own steady-state weights and ensuing time traces of k_{TX} and ξ are generated independently of each other. Only when ξ stays at unity over the whole simulation time, mRNAs are produced. The lifetime distributions of the active and inactive gene states are respectively given by $\psi_{on}(t) = k_a e^{-k_a t}$ with $k_a = 0.34$ min^{-1} and $\psi_{off}(t) = t^{a-1}e^{-t/b} / \Gamma(a)b^a$ with $a = 6.66$ and $b = 9.73$ min. The (i+1)-th mRNA creation time, t_{i+1}^c, is sampled from

$k_{TX}(t_i^c)e^{-tk_{TX}(t_i^c)}$, where $k_{TX}(t_i^c)$ designates the value of k_{TX} at the *i*-th mRNA creation time, t_i^c. When k_{TX} undergoes a transition before a creation event is completed, the incomplete creation event is discarded and the new creation starts at the time of the transition. To calculate the mRNA number power spectrum, we use the same algorithm described in S6 Text, Section C.
