# Supplementary material for: Frequency spectrum of chemical fluctuation: A probe of reaction mechanism and dynamics
Source: PLoS Comput Biol. 2019 Sep 16;15(9):e1007356. doi: 10.1371/journal.pcbi.1007356 (PMC6762214; doi:10.1371/journal.pcbi.1007356)
Supplement: S10 Text — (PDF) [file pcbi.1007356.s010.pdf]

Supplementary Text 10 | Derivation of Eq 12.

Here, we provide a detailed derivation of Eq 12. The mean-scaled time correlation function of the translation rate, $R_{TL}(=k_{TL}m)$, can be written as [1]

$$\frac{\langle \delta R_{TL}(t)\delta R_{TL}(0)\rangle}{\langle R_{TL}\rangle^2} = \eta_{k_{TL}}^2 \phi_{k_{TL}}(t) + \eta_m^2 \phi_m(t) + \eta_{k_{TL}}^2 \eta_m^2 \phi_{k_{TL}}(t)\phi_m(t) \quad \text{(S10-1)}$$

unless k_{TL} is correlated with *m*. When $\phi_{k_{TL}}(t)$ relaxes much slower than $\phi_m(t)$, Eq S10-1 can be simplified as

$$\frac{\langle \delta R_{TL}(t)\delta R_{TL}(0)\rangle}{\langle R_{TL}\rangle^2} \cong \eta_{k_{TL}}^2 + (1+\eta_{k_{TL}}^2)\eta_m^2 \phi_m(t) \quad \text{(S10-2)}$$

The Fourier transform of Eq S10-2 is given by

$$\begin{aligned} \frac{S_{R_{TL}}(\omega)}{\langle R_{TL}\rangle^2} &= \eta_{k_{TL}}^2 \delta(\omega) + (1+\eta_{k_{TL}}^2)\eta_m^2 \tilde{\phi}_m(\omega) \\ &= \eta_{k_{TL}}^2 \delta(\omega) + (1+\eta_{k_{TL}}^2)\frac{S_m(\omega)}{\langle m\rangle^2} \end{aligned} \quad \text{(S10-3)}$$

For nonzero frequency $(\omega > 0)$, Eq 12 can be obtained by substituting Eq S10-3 into Eq S5-1and noting that $\langle R_{TL}\rangle = \langle k_{TL}\rangle\langle m\rangle$.

Reference

1. Lim YR, Kim J-H, Park SJ, Yang G-S, Song S, Chang S-K, et al. Quantitative Understanding of Probabilistic Behavior of Living Cells Operated by Vibrant Intracellular Networks. Phys Rev X. 2015;5(3):031014.
