# Supplementary material for: Frequency spectrum of chemical fluctuation: A probe of reaction mechanism and dynamics
Source: PLoS Comput Biol. 2019 Sep 16;15(9):e1007356. doi: 10.1371/journal.pcbi.1007356 (PMC6762214; doi:10.1371/journal.pcbi.1007356)
Supplement: S1 Fig — (PDF) [file pcbi.1007356.s011.pdf]

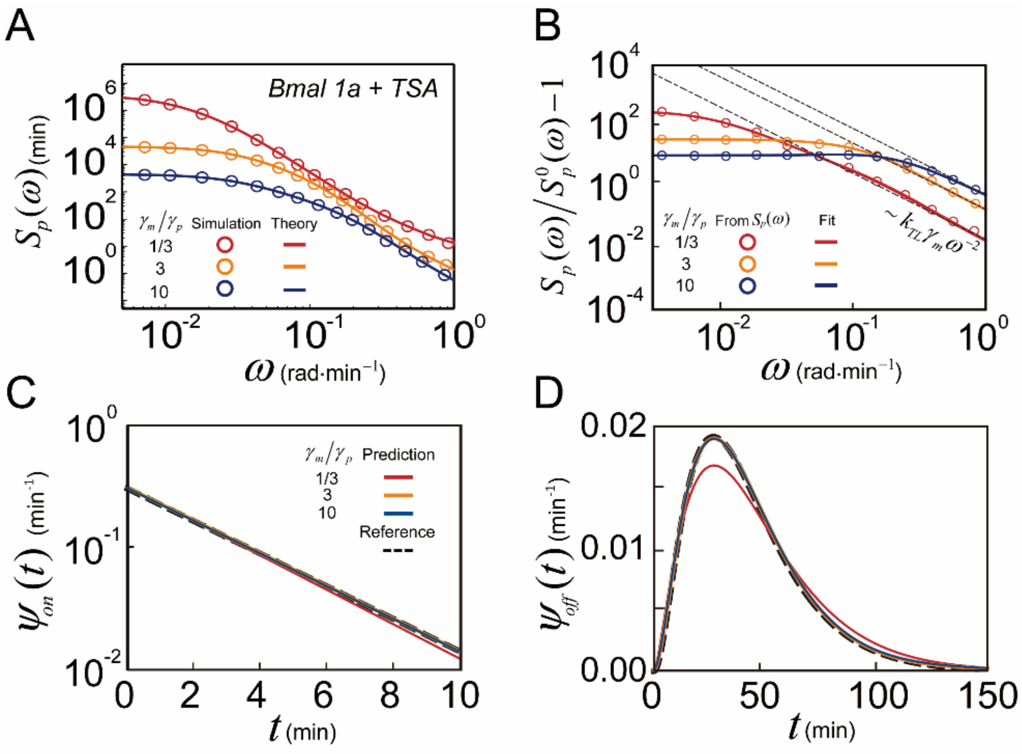

Fig S1. Power spectrum analysis of *Bmal 1a* + TSA gene expression. (A) (circles) Power spectrum calculated from the protein number time traces obtained by the simulation of the gene expression network shown in Fig 3A and 3B with use of the reference values of the rate parameters reported in reference [1] for luciferase expression under *Bmail 1a* in mice fibroblast cells. The authors in reference [1] investigated the special case where the lifetime of the luciferase is adjusted to be one-third of its mRNA lifetime. In our simulation, we also conduct the simulation of the gene expression network model for the usual case where the protein lifetime is longer than the mRNA lifetime. It is known that, in the presence of TSA, the number of intermediate reaction steps composing gene activation or the mean lifetime of the inactive gene state decreases. To analyze this shortening, Naef and co-workers propose that the number of gene activation steps is three, instead of seven. By using the reference value for the rate parameters other than the protein decay rate, we conduct simulations for the gene expression network shown in Fig 3A and 3B to obtain the time traces of mRNA and protein numbers. (line) Prediction of our theory for the same gene expression network model. (B) Quantitative analysis

of the simulation data for $S_p(\omega)\big/S_p^0(\omega)-1$. $S_p^0(\omega)$ is defined as $S_p^0(\omega)=2\langle R_{TL}\rangle\big/\left(\omega^2+\gamma_p^2\right)$. Given the value of γ_p, one can estimate the value of the mean translation rate, $\langle R_{TL}\rangle$, from the mean protein number, $\langle p\rangle$, by $\langle R_{TL}\rangle=\gamma_p\langle p\rangle$. In the quantitative analysis of the data, we have used Eq S7-2, which is obtained for the gene expression network model in Fig 3A with the Poisson gene deactivation process and the non-Poisson gene activation process with the reaction waiting time distribution given by a gamma distribution. The values of the extracted parameter are given by $a=2.86$, $b=15.43$, and $\tau_{on}=4.37\,\text{min}$ for $\gamma_m\big/\gamma_p=1/3$, $a=2.78$, $b=15.13$, and $\tau_{on}=3.21\,\text{min}$ for $\gamma_m\big/\gamma_p=10$. (C, D) Protein number power spectrum results. Note that $\psi_{on}(t)$ extracted from this analysis is approximately the same as $\psi_{on}(t)$ shown in Fig S2E, but $\psi_{off}(t)$ extracted from this analysis has a smaller mean than $\psi_{off}(t)$ extracted from the analysis shown in Fig S2F.

Reference

1. Zoller B, Nicolas D, Molina N, Naef F. Structure of silent transcription intervals and noise characteristics of mammalian genes. Mol Syst Biol. 2015;11(7):823.
