# Supplementary material for: Frequency spectrum of chemical fluctuation: A probe of reaction mechanism and dynamics
Source: PLoS Comput Biol. 2019 Sep 16;15(9):e1007356. doi: 10.1371/journal.pcbi.1007356 (PMC6762214; doi:10.1371/journal.pcbi.1007356)
Supplement: S2 Fig — (PDF) [file pcbi.1007356.s012.pdf]

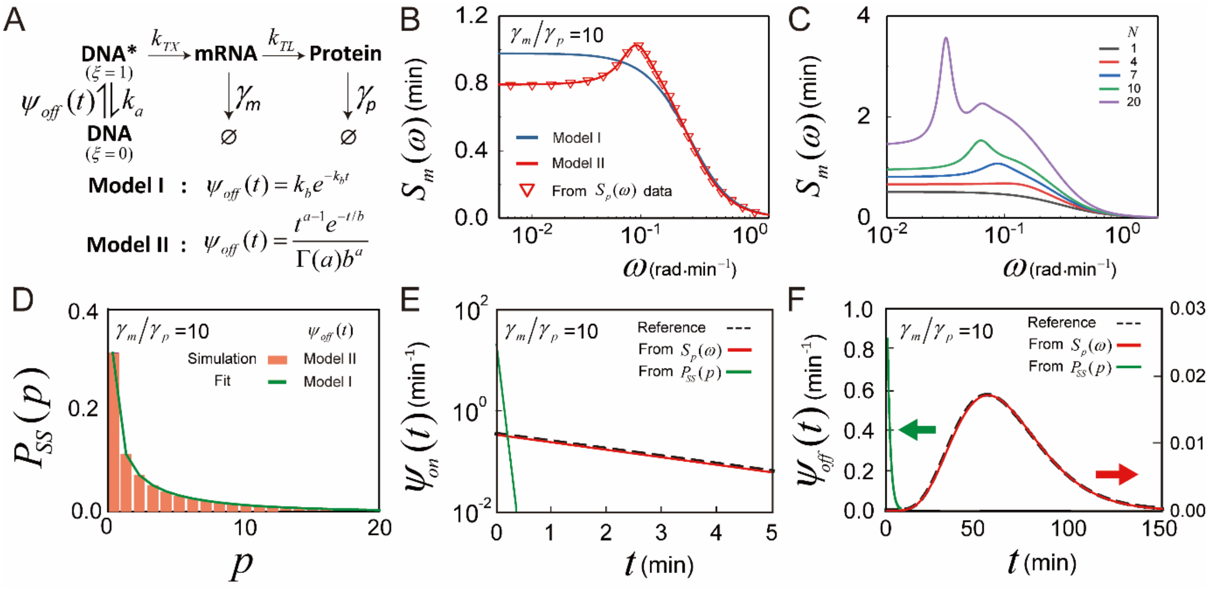

Fig S2. Quantitative analysis of the protein number frequency spectrum. (A) Gene expression network models with two different gene activation processes: Poisson process (Model I) and non-Poisson process with a gamma distributed waiting time (Model II). When the gene activation process composed of N consecutive Poisson processes with rate constants given by the same constant, k, the lifetime distribution, $\psi_{off}(t)$, of the inactive gene state is given by a gamma distribution, $t^{a-1}e^{-t/b}/b^a\Gamma(a)$, with a and b being N and k^{-1}, respectively. (B) (triangles) mRNA number frequency spectrum corresponding to the data shown by the blue triangles in Fig 2F. (blue line) the best fitted result of Model I, (red line) the best fitted result of Model II. (C) Dependence of $S_m(\omega)$ on the number, N, of the consecutive reaction steps comprising the gene activation process. As N increases, the peak position shifts to the lower frequency, and the peak shape gets sharper. (D) Steady-state distribution of the protein number. (histogram) Simulation results for the gene expression network model in Fig 2A and B. (green line) the best fitted result of Model I. (circles) (E, F) Lifetime distributions, $\psi_{on}(t)$ and $\psi_{off}(t)$, of the active and inactive gene states, extracted from the frequency spectrum analysis by Model I (blue line) and Model II (red line), and from

the steady-state protein number distribution by Model I (green line). $\psi_{on}(t)$ and $\psi_{off}(t)$, which were extracted from the frequency spectrum analysis with use of Model II, are in excellent agreement with the reference model in Fig 2A and B. The steady state distribution of the protein number in Fig S2D can be explained by Model I (green line). However, $\psi_{on}(t)$ and $\psi_{off}(t)$, extracted by Model I from the steady-state protein number distribution, greatly deviate from the data.
