# Supplementary material for: Frequency spectrum of chemical fluctuation: A probe of reaction mechanism and dynamics
Source: PLoS Comput Biol. 2019 Sep 16;15(9):e1007356. doi: 10.1371/journal.pcbi.1007356 (PMC6762214; doi:10.1371/journal.pcbi.1007356)
Supplement: S3 Fig — (PDF) [file pcbi.1007356.s013.pdf]

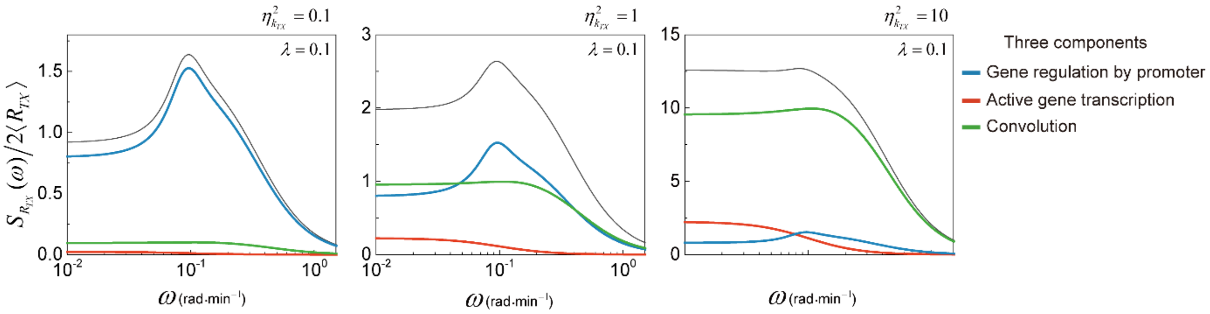

Fig S3. Three components in the power spectrum of the transcription rate. (black line) The value of $\Delta_m(\omega) = \sum_{i=1}^{3} \Delta_m^{(i)}(\omega)$ or $\eta^2_{k_{TX}}$ with $S_m^0(\omega) = 2\langle m\rangle \gamma_m \big/ (\omega^2 + \gamma_m^2)$. The value of λ is here set to be 0.1 min^{-1}. (colored lines) Three components of the power-spectrum: $\langle R_{TX}\rangle \tilde{S}_\xi(\omega)\big/2$ originating from the gene regulating dynamics of promoter (blue), $\langle R_{TX}\rangle \tilde{S}_{k_{TX}}(\omega)\big/2$ originating from the active gene transcription dynamics (red), and their convolution $\langle R_{TX}\rangle \tilde{S}_\xi(\omega) * \tilde{S}_{k_{TX}}(\omega)\big/2$ (green). See Eqs 11 or S8-4. Unlike the case shown in Fig 4C, the peak originating from the promoter fluctuation diminishes with $\eta^2_{k_{TX}}$ because a fast fluctuation in k_{TX} with a rate λ comparable to or larger than the promoter fluctuation frequency, $\omega_{peak} = 2\pi\big/(\tau_{on} + \tau_{off}) \cong 0.1$ rad·s^{-1}, effectively filters out the contribution of the promoter fluctuation in $\langle R_{TX}\rangle \tilde{S}_\xi(\omega) * \tilde{S}_{k_{TX}}(\omega)\big/2$.
