# Supplementary material for: Frequency spectrum of chemical fluctuation: A probe of reaction mechanism and dynamics
Source: PLoS Comput Biol. 2019 Sep 16;15(9):e1007356. doi: 10.1371/journal.pcbi.1007356 (PMC6762214; doi:10.1371/journal.pcbi.1007356)
Supplement: S4 Fig — (PDF) [file pcbi.1007356.s014.pdf]

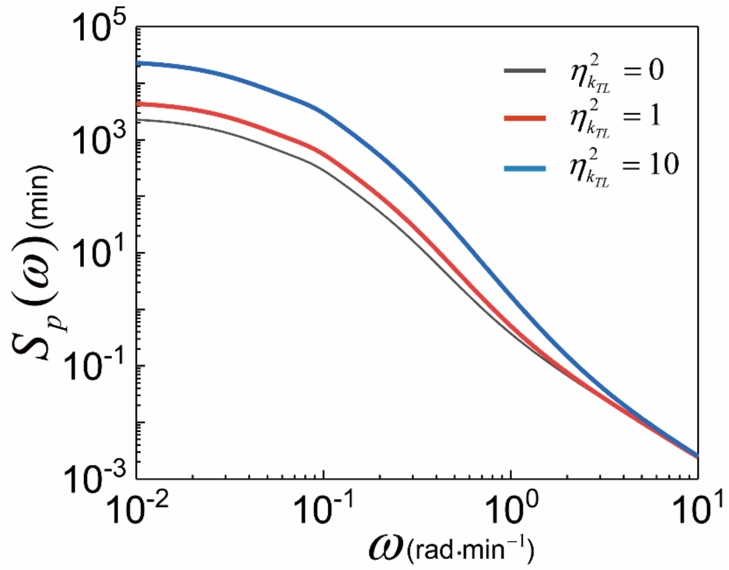


**Fig S4. Effect of the cell-to-cell heterogeneity in translation rate coefficient, $k_{TL}$, on the protein number power spectrum, $S_p(\omega)$.** The relevant expression of $S_p(\omega)$ is reproduced here: $S_p(\omega) = S_p^0(\omega)\left[1 + \langle k_{TL}\rangle(1+\eta^2_{k_{TL}})S_m(\omega)/2\langle m\rangle\right]$, which is equivalent to Eq 12. $S_p^0(\omega)$ and $S_m(\omega)$ denote the protein number power spectrum without any fluctuation in translation rate, $R_{TL}(=k_{TL}m)$ and the mRNA number power spectrum, respectively. As shown in the equation given above, an increase of the relative variance, $\eta^2_{k_{TL}}$, of $k_{TL}$ amplifies the contribution of the mRNA number power spectrum to the protein number power spectrum. As a result, the protein number power spectrum increases with $\eta^2_{k_{TL}}$. Here, the protein number power spectrum with $\eta^2_{k_{TL}} = 0$ is the same as that given in Fig 3C when the value of $\gamma_m/\gamma_p$ is equal to 10. However, the protein number power spectrum is not essentially affected by $\eta^2_{k_{TL}}$ over the high frequency range in which $S_p^0(\omega)$ dominates the other term, $\langle k_{TL}\rangle(1+\eta^2_{k_{TL}})S_p^0(\omega)S_m(\omega)/2\langle m\rangle$.
