# Supplementary material for: Frequency spectrum of chemical fluctuation: A probe of reaction mechanism and dynamics
Source: PLoS Comput Biol. 2019 Sep 16;15(9):e1007356. doi: 10.1371/journal.pcbi.1007356 (PMC6762214; doi:10.1371/journal.pcbi.1007356)
Supplement: S5 Fig — (PDF) [file pcbi.1007356.s015.pdf]

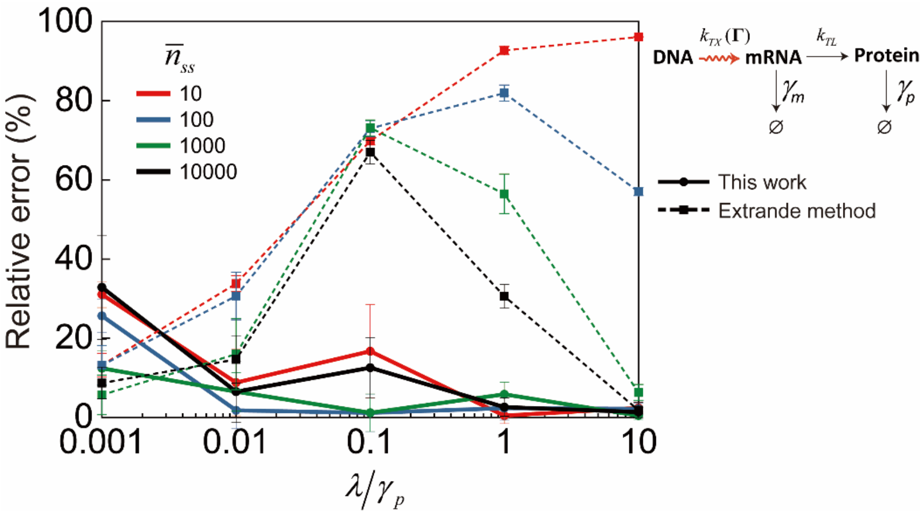

Fig S5. Performance of the current method of simulating reaction events occurring with stochastically fluctuating rate in comparison with the Extrande method. In order to compare our method for simulating reaction events, which occur with stochastically fluctuating rate, with the Extrande method [1], we performed the stochastic simulation for the gene expression network, which is exactly the same as that shown in Fig 1B of reference [1]. In this simulation, the transcription rate, k_{TX}, is modeled as $k_{TX}(\Gamma)/\gamma_p = \exp(\Gamma)/\langle\exp(\Gamma)\rangle$, where Γ denotes an Ornstein-Uhlenbeck process characterized by zero mean and time autocorrelation function given by $\langle\Gamma(t)\Gamma(t')\rangle = \langle\Gamma^2\rangle\exp(-\lambda\,|t-t'|)$. In the steady state, the average number, $\langle n(t)\rangle$, of proteins is calculated from 100 protein traces at each $100\gamma_p^{-1}(=\Delta t)$ interval until the simulation time reaches $T = 10^4\gamma_p^{-1}$. With values of $\langle n(t)\rangle$ at 100 time points, we calculate the relative error as $\left|\sum_{i=1}^{N=100}\langle n(i\Delta t)\rangle/N - \bar{n}_{ss}\right|/\bar{n}_{ss}$, where $\bar{n}_{ss}$ denotes the theoretical reference for the steady-state mean protein number given by $\bar{n}_{ss} = \langle k_{TX}(\Gamma)\rangle k_{TL}/\gamma_m\gamma_p\,(= k_{TL}/\gamma_m)$. In this simulation, the values of $\langle\Gamma^2\rangle$, γ_m/γ_p, and k_{TL}/γ_p are given by $\langle\Gamma^2\rangle = 5$, $\gamma_m/\gamma_p = 4$, and $k_{TL}/\gamma_p = \bar{n}_{ss}\gamma_m/\langle k_{TX}(\Gamma)\rangle = \bar{n}_{ss}\gamma_m/\gamma_p$, respectively. The error bar represents the standard deviation of the bootstrap distribution, which is here constructed by randomly taking one

thousand samples from the set, $\{\langle n(i\Delta t)\rangle \,|\, 1 \le i \le 100\}$. When the value of λ/γ_p is larger than roughly 0.003 or 0.004, our method shows a faster convergence than the Extrande method. We also confirmed that the relative error of our method finally becomes negligible when a large number of protein traces are used. In our case with Intel i7-6700K 4.00GHz quad-core processor, the most time-consuming simulation with $\lambda/\gamma_p = 10^{-3}$ and $\overline{n}_{ss} = 10^4$ takes about ten minutes. When $\lambda/\gamma_p = 10$ and $\overline{n}_{ss} = 10$, the simulation takes about one minute with the same processor.

Reference

1. Voliotis M, Thomas P, Grima R, Bowsher CG. Stochastic simulation of biomolecular networks in dynamic environments. PLoS Comput Biol. 2016;12(6):e1004923.
